# Cryo-EM structures of human ABCB7 reveal the molecular basis of mitochondrial matrix heme export

**DOI:** 10.1038/s42003-026-10223-x

**Published:** 2026-05-09

**Authors:** Seulgi Ju, Seung Hun Choi, Hyeon You Lee, Mi Sun Jin

**Affiliations:** 1 Department of Life Sciences, Gwangju Institute of Science and Technology, Gwangju, South Korea; 2 Integrated Institute of Biomedical Research, Gwangju Institute of Science and Technology, Gwangju, South Korea

**Keywords:** Cryoelectron microscopy, Cryoelectron microscopy

## Abstract

ATP-binding cassette transporter subfamily B member 7 (ABCB7) is a mitochondrial ATP-driven pump essential for cytosolic iron–sulfur (Fe-S) cluster biogenesis and cellular iron homeostasis. Mutations in ABCB7 are linked to X-linked sideroblastic anemia with ataxia (XLSA/A). Here, we demonstrate that the ATPase activity of ABCB7 is stimulated by iron and cobalt protoporphyrin IX (hemin and CoPP) in the presence of glutathione (GSH), highlighting an additional role for ABCB7 as a metalloporphyrin exporter. Using single-particle cryo-electron microscopy, we determine the structures of human ABCB7 in multiple functional states at resolutions of up to 2.3 Å. Our structures reveal a putative substrate-binding cavity that accommodates two stacked CoPP molecules conjugated by two GSH cysteine thiols. The conserved residue F426 controls substrate entrapment and release as a molecular gate. We further show that at high substrate concentrations excess CoPP easily partitions into the lipid bilayer, where the hydrophobic environment stabilizes the porphyrin macrocycle and limits the aggregation or redox reactivity that is prone to occur with free porphyrins in aqueous solution. Finally, our structure analyses rationalize the pathogenic effect of the E433K mutation associated with XLSA/A disease.

## Abstract (web summary)

Cryo-EM structures of human ATP-binding cassette transporter 7 (ABCB7) reveal a glutathione-dependent mechanism for metalloporphyrin transport across the mitochondrial inner membrane.

## Introduction

The ATP-binding cassette (ABC) transporters are a large and diverse family of membrane proteins that use the energy derived from ATP hydrolysis to translocate a wide range of molecular cargos across cell membranes^1,2^. These transporters are essential for numerous biological processes, including nutrient uptake, detoxification, lipid transport, ion homeostasis, antigen presentation, and ion channel regulation^1,3^. To date, 49 ABC transporter genes have been identified in humans and classified into seven subfamilies (ABCA to ABCG). The fundamental architecture of a transporter consists of two transmembrane domains (TMDs) and two nucleotide-binding domains (NBDs) interconnected by short coupling helices. These domains work in concert to facilitate substrate translocation through ATP-dependent conformational changes^4,5^.

There are four members of mitochondrial ABC subfamily B (ABCB6, ABCB7, ABCB8, and ABCB10), of which one, ABCB7, is located in the inner membrane of mitochondria and plays an essential role in cytosolic Fe-S cluster biogenesis and cellular iron homeostasis^6,7^. It is a Type IV half-transporter containing one TMD and one NBD, which assembles into a homodimeric complex^8^. It is a structural and functional homolog of yeast Atm1, the first identified mitochondrial ABC transporter, sharing 49% sequence identity with it^9,10^. Loss-of-function mutations in Atm1/ABCB7 result in accumulation of iron within mitochondria, oxidative stress, and severe growth defects^10–15^. Atm1/ABCB7 is also crucial for early embryonic development, B cell maturation, tRNA thiolation, hematopoiesis, and inhibition of both apoptotic and non-apoptotic cell death^6,16–21^. Defects in ABCB7 are associated with X-linked sideroblastic anemia with ataxia (XLSA/A), an inherited disorder characterized by disrupted heme biosynthesis, mitochondrial iron accumulation and defective Fe-S cluster maturation^14,21–24^. More recently, whole-genome sequencing has identified a novel ABCB7 mutation associated with X-linked congenital cerebellar ataxia, further underscoring the critical role of ABCB7 in early motor neuron development^25^.

Atm1/ABCB7 proteins are known to be involved in the transport of various molecular species across membranes. Thiol compounds, such as glutathione (GSH), glutathione persulfide (GSSG), and dithiothreitol (DTT) have been proposed as potential substrates from studies of bacterial, yeast, and plant ABCB7 homologues^26–29^. In vitro studies have further suggested that GSH-conjugated Fe-S clusters are substrates for Atm1 and ABCB7^30–32^. Bacterial and zebrafish ABCB7 homologues have also been reported to function as heavy metal transporters involved in cellular detoxification^29,33,34^. X-ray crystallographic and cryo-electron microscopy (cryo-EM) analyses have further enhanced our understanding of the molecular mechanisms of Atm1-like transporters^29,32,35–39^. However, the mechanism of substrate binding and transport by human ABCB7 remains largely unknown because structural information is currently limited to a single functional state (i.e., the AMP-PNP-bound form)^35^.

Here, we identify iron and cobalt protoporphyrin IXs (hemin and CoPP) as substrates of human ABCB7, and show that their ATPase activities are selectively stimulated by these metalloporphyrins in the presence of GSH. Using cryo-EM, we determine structures of ABCB7 in multiple functional states; apo (3.0 Å), CoPP-bound (3.4 Å), CoPP and ADP·VO_4_-bound (2.8 Å), and ATP-bound (2.3 Å). Together with homology modelling of a transient outward-facing conformation, our structures delineate the substrate recognition and transport mechanism of ABCB7 at near-atomic resolution. Finally, our data provide structural insights into the disease mechanism of the XLSA/A-linked E433K mutation.

## Results

### The ATPase activity of human ABCB7 is stimulated by iron and cobalt protoporphyrin IX in the presence of GSH

Recent studies have reported that two ABCB7-related transporters, ABCB6 and ABCB10, are involved in porphyrin transport^40–43^. In particular, ABCB6 transports metal-centered porphyrin as a complex with two molecules of glutathione (GSH), whereas metal-free porphyrin is transported in its unbound form^40,41^. Since ABCB7 shares 42% and 29% sequence identity, respectively, with these transporters, we hypothesized that it might similarly mediate the active transport of porphyrins from the mitochondrial matrix to the intermembrane space (Supplementary Fig 1). This hypothesis is supported by the fact that ABCB7 co-localizes and forms a stable complex with ferrochelatase (FECH), the enzyme responsible for incorporating Fe²⁺ into protoporphyrin IX in the final step of heme synthesis^19,20^.

**Fig. 1:**
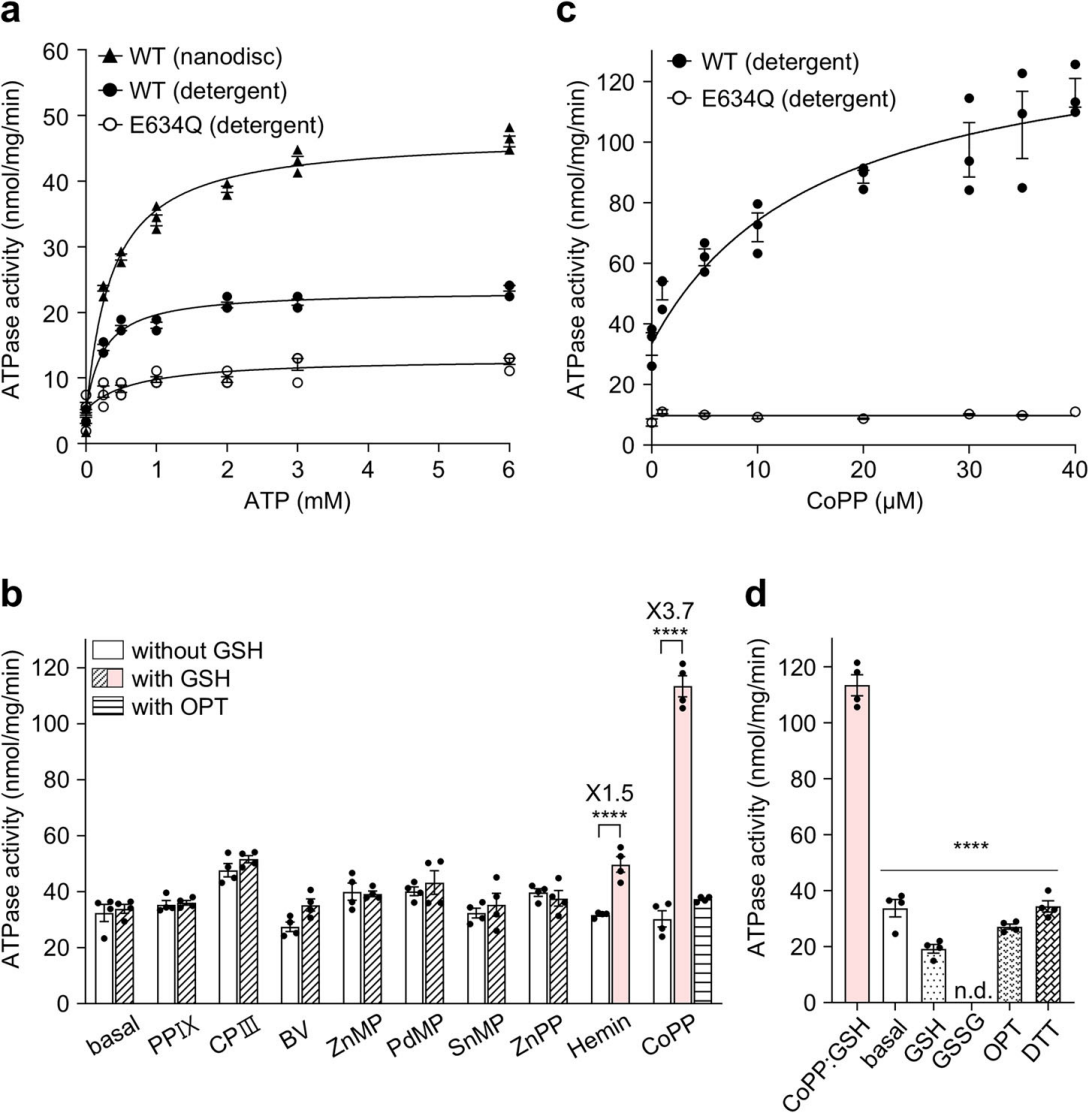
ATPase activity of human ABCB7. **a** ATPase activities (NADH-coupled assay) of wild-type ABCB7 and the catalytically impaired E634Q mutant. Nonlinear regression fitting of the Michaelis–Menten equation yielded apparent kinetic parameters for wild-type ABCB7 of K_m_ = 0.7 ± 0.2 mM and V_max_ = 43 ± 0.8 nmol mg⁻¹ min⁻¹ in nanodiscs, and K_m_ = 0.2 ± 0.03 mM and V_max_ = 18.5 ± 0.2 nmol mg⁻¹ min⁻¹ in LMNG micelles. The E634Q mutant displayed corresponding values of K_m_ = 0.9 ± 0.4 mM and V_max_ = 7.9 ± 0.7 nmol mg⁻¹ min⁻¹ in detergent. Data points represent mean ± standard error of the mean (SEM) of four independent measurements. **b** ATPase activities (molybdate phosphate assay) of detergent-purified ABCB7 in the presence or absence of 40 μM porphyrins (*n* = 4). Assays were conducted with and without 1 mM GSH (or OPT). ****p  <  0.0001 (two-way ANOVA, Tukey’s test). All ATP hydrolysis data were corrected for background activity measured in the absence of protein and substrates. Protoporphyrin IX (PPⅨ), coproporphyrinogen III (CPⅢ), biliverdin (BV), zinc(II) mesoporphyrin IX (ZnMP), palladium(II) mesoporphyrin IX (PdMP), tin mesoporphyrin IX (SnMP), zinc protoporphyrin (ZnPP), hemin (iron(III) protoporphyrin IX chloride), and cobalt(III) protoporphyrin IX chloride (CoPP). **c** ATPase activities of wild-type ABCB7 and the E634Q mutant as a function of CoPP concentration (*n* = 3). The GSH concentration was kept at 1 mM. The kinetic values for CoPP:GSH were K_m_ 16 ± 5 μM and V_max_ 106 ± 14 nmol mg⁻¹ min⁻¹. **d** ATPase activities of ABCB7 in the presence of GSH, GSH derivatives, and DTT (*n* = 4). For comparison, the basal and CoPP:GSH-stimulated ATPase activities of ABCB7 (adapted from **b**) are also shown. Statistical significance was evaluated using two-way ANOVA followed by Dunnett’s multiple comparisons test. n.d. not detected.

To test this idea, we overexpressed human ABCB7 in an insect cell system and purified it by affinity and size-exclusion chromatography. Using purified protein in lauryl maltose neopentyl glycol (LMNG), we initially attempted microscale thermophoresis (MST) measurements and liposome-based in vitro transport assays with various porphyrins to examine whether ABCB7 bound and transported them as putative substrates^44^. However, these approaches did not yield reliable results, as metal-free porphyrins showed no detectable interaction with ABCB7, whereas metalloporphyrins exhibited strong nonspecific interactions with detergent micelles and liposome membranes that resulted in high background signals^45,46^. We therefore turned to ATPase assays under various conditions, as many ABC transporters have been reported to couple ATP hydrolysis to substrate transport (Fig. 1a–d and Supplementary Fig 2)^3^. Kinetic analysis of detergent-purified ABCB7 revealed a K_m_ for ATP of 0.2 ± 0.03 mM and a maximal basal turnover rate of 18 ± 0.2 nmol/mg/min (Fig. 1a). When reconstituted into nanodiscs, ABCB7 had a comparable K_m_ for ATP but a 2.3-fold higher V_max_ of 43 ± 0.8 nmol/mg/min. We further demonstrated that of various porphyrins tested (Supplementary Fig 2a–i), iron protoporphyrin IX (hemin, the oxidized version of heme with a chloride ligand) and cobalt protoporphyrin IX (CoPP) stimulated the ATPase activity of ABCB7 by approximately 1.5- and 3.7-fold, respectively, but only in the presence of GSH (Fig. 1b). Under these conditions, the measured K_m_ and V_max_ values for CoPP were 16 ± 5 μM and 106 ± 14 nmol/mg/min, respectively (Fig. 1c). Other metalloporphyrin scaffolds and metal-free analogs lacked a stimulatory effect (Fig. 1b and Supplementary Fig 2). In contrast, the catalytically inactive E634Q mutant had little basal ATPase activity and was not stimulated by CoPP:GSH (Fig. 1a, c). Notably, when GSH was replaced by ophthalmic acid (OPT) in which the cysteine is substituted by 2-aminobutyrate, CoPP failed to stimulate ABCB7 activity (Fig. 1b). Furthermore, unlike Atm1-type transporters, whose ATPase activity is stimulated by thiol compounds alone including GSH, its oxidized form and DTT (Supplementary Fig 2j–m), the ATPase activity of human ABCB7 was not stimulated by such compounds (Fig. 1d)^29,36,38^. These results indicate that the substrate specificity of the latter differs fundamentally from that of its non-mammalian homologs. Collectively, our findings identify ABCB7 as a previously unrecognized mitochondrial metalloporphyrin exporter, in which the central metal within the protoporphyrin scaffold is indispensable for complex formation with ABCB7 through interaction with the cysteine thiol group of GSH.

### Overall architecture of ABCB7 in an inward-facing conformation

**Fig. 2:**
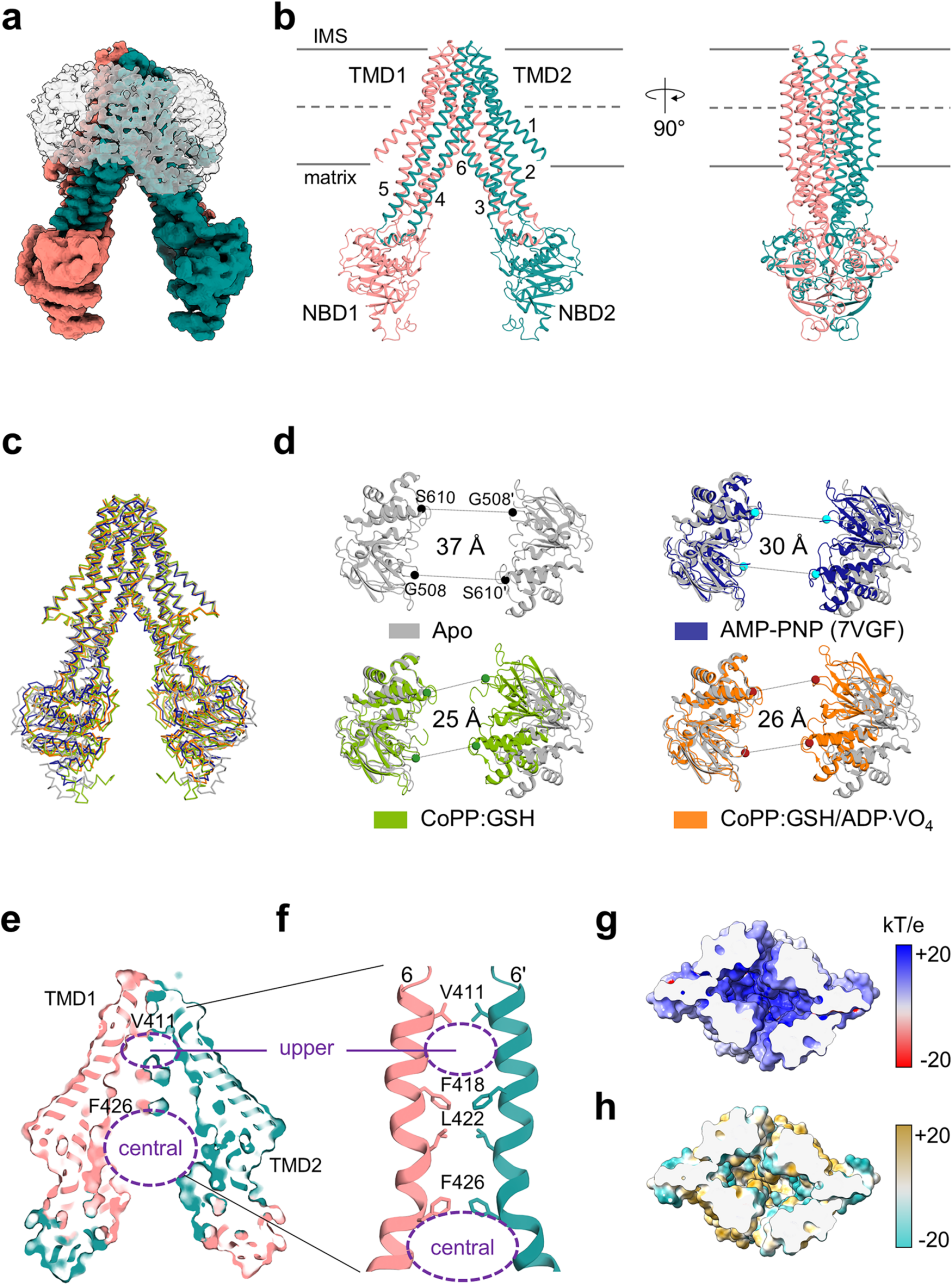
Overall structures of apo ABCB7 and substrate-bound ABCB7. **a** Cryo-EM map of homodimeric ABCB7 in the apo inward-facing state, with monomers shown in salmon and deep teal. **b** Overall structure of apo ABCB7. **c** Superimposed structures of ABCB7 in the inward-facing open conformation: apo (grey), AMP-PNP–bound (PDB ID 7VGF, blue), CoPP:GSH–bound (green), and CoPP:GSH/ADP·VO₄–bound (orange). For clarity, bound substrate and nucleotide molecules are omitted. **d** Comparison of NBDs in various inward-facing states. Cα distances between the conserved G508 (Walker A motif) and S610 (signature motif) are indicated. For comparison, the position of one NBD in the substrate- (or AMP-PNP–bound) state is shown relative to the other NBDs in the apo conformation. **e** Surface slab view of apo ABCB7. **f** The residues of TM 6 involved in stabilization of the inward-facing conformation are shown as sticks. **g** Surface electrostatic potential of the substrate-binding site shown in bottom view. The potential was calculated using the APBS/PDB2PQR software suite at pH 7.5 (http://www.poissonboltzmann.org). **h** Hydrophobic surface representation of the substrate-binding site in bottom view. The molecular surface illustrates pocket hydrophobicity (cyan, hydrophilic; yellow/brown, hydrophobic).

To validate our functional characterization of human ABCB7 (Fig. 1) and dissect its transport mechanism, we prepared various cryo-EM samples combining putative substrates and different nucleotide states (Supplementary Fig. S3–S14 and Supplementary Table 1). Cryo-EM analysis demonstrated that apo ABCB7 has an inverted V-shaped homodimeric architecture with the topology of a type IV eukaryotic ABC exporter (Fig. 2a, b)^8^. Each monomer consists of a TMD containing six transmembrane helices (TM1–6) and a cytosolic NBD. TMD1 and TMD2 adopt a domain-swapped arrangement in which TM4 and TM5 from each half are exchanged with the other half. Structural comparisons revealed that our apo, substrate-bound, and substrate/ADP·VO_4_-bound structures and the previously reported AMP-PNP–bound form (PDB ID 7VGF)^35^, were in highly similar inward-facing states (Fig. 2c). Nevertheless, these states exhibit different extents of NBD separation. In the apo state, the two ATP-binding sites are separated by ~37 Å (Fig. 2d); nucleotide binding then brings the NBDs closer together, reducing the inter-NBD distance to ~30 Å. Substrate binding induces a further inward rotation of the NBDs, decreasing their separation to ~25 Å. In light of the substrate-stimulated ATPase activity of ABCB7 shown in Fig. 1c, this substrate-induced closure likely primes the transporter for ATP binding by aligning optimally the Walker A and signature motifs of opposing NBDs.

In the apo state, ABCB7 has two separate substrate-binding cavities—central and upper—arranged along the translocation pathway (Fig. 2e). The three hydrophobic residues of TM6 (F418, L422, and F426) are positioned close to their symmetric counterparts, defining the boundary between the central and upper cavities (Fig. 2f). This narrow constriction (gate 1), with an inter-residue distance of 3.5–5.8 Å, probably governs substrate movement from the central to the upper cavity during export. At the apex of the upper cavity, the V411 residue pair forms a tight hydrophobic seal (gate 2) that blocks access to the extracellular medium, thereby establishing a two-gate configuration. This two-cavity arrangement is distinct from that of Atm-1 proteins, which typically feature a single continuous translocation tunnel^32,37–39^. Further structure analysis revealed that the central cavity is sufficiently large ( ~ 2100 Å^3^) to accommodate bulky substrates, such as the GSH-conjugated metalloporphyrins or Fe-S clusters^47^. Its surface is characterized by a predominantly positive charge at the inward entrance and a hydrophobic profile at the cavity apex (Fig. 2g, h). This environment would seem to favor the accommodation of hydrophobic porphyrin rings bearing acidic propionic groups, but to be unfavorable for cationic metal centers due to charge repulsion. Structure analysis thus suggests that the transporter requires GSH as a cofactor to shield the metal ion charge and thereby facilitate stable substrate binding.

### Substrate recognition

**Fig. 3:**
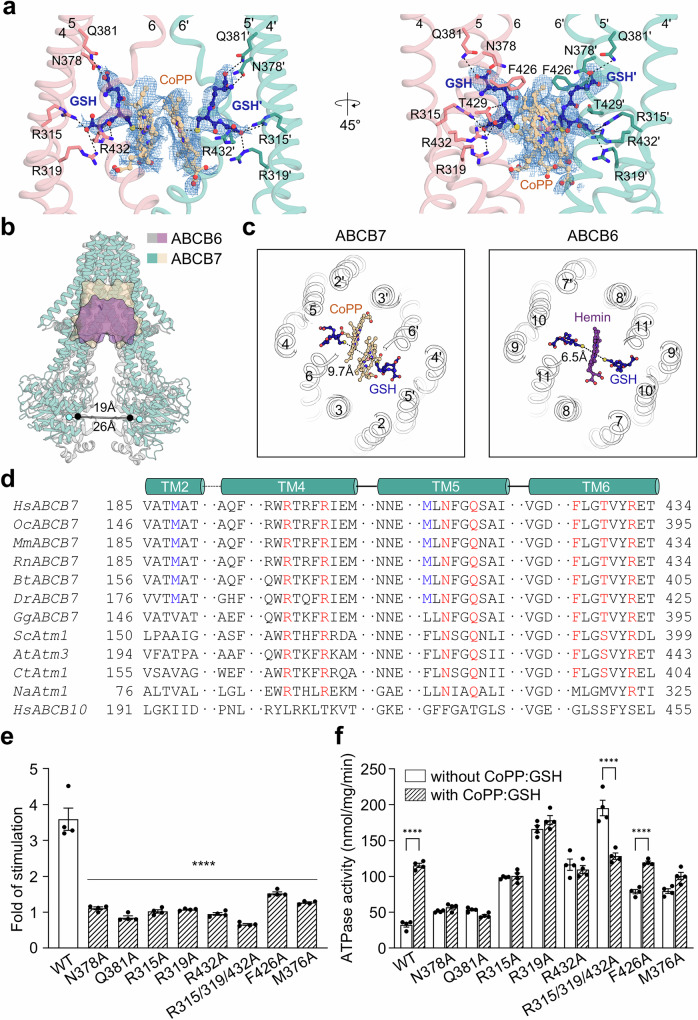
The substrate binding site. **a** Close-up view of the binding site of the CoPP:GSH complex. Residues interacting with bound substrate were identified by LigPlot+ software and are shown as sticks. The EM density maps for bound CoPP and GSH were contoured at 2.2 σ and 4 σ, respectively. **b** Comparison of the substrate binding cavities of human porphyrin transporters ABCB6 and ABCB7. The cavities are colored purple and wheat, respectively. **c** Top views of the substrate binding sites of ABCB6 and ABCB7. The distances between the two GSH molecules coordinating porphyrin are indicated. **d** Sequence alignments of human ABCB7 homologs. Residues in the central cavity that interact with the CoPP:GSH substrate are highlighted in red; transmembrane residues contacting CoPP within the lipid bilayer are highlighted in blue (see also Fig. 4). *Hs, Homo sapiens; Oc,Oryctolagus cuniculus; Mm, Mus musculus; Rn, Rattus norvegicus; Bt, Bos taurus; Dr, Danio rerio; Gg, Gallus gallus; Sc, Saccharomyces cerevisiae; At, Arabidopsis thaliana; Ct, Chaetomium thermophilum; Na, Novosphingobium aromaticivorans*. **e**,**f** ATPase activities of substrate-binding mutants measured in the presence or absence of 40 µM CoPP and 1 mM GSH. In (**e**), substrate-stimulated activity is expressed as fold change relative to the basal activity of each mutant. Data represent mean ± SEM (*n* = 4). ****p  <  0.0001 (two-way ANOVA Dunnett’s test).

In the presence of CoPP:GSH, both with and without ADP·VO₄, an additional electron density was observed within the central cavity corresponding to the bound CoPP:GSH complex (Fig. 3a). The two structures had nearly identical conformations (Cα r.m.s.d.< 1.3 Å), with residues contacting the CoPP:GSH complex adopting the same arrangement (Fig. 2c, d). Intriguingly, in the CoPP:GSH/ADP·VO₄-bound dataset, only a small subset of particles (8.9%) adopted the expected occluded conformation with a closed NBD dimer (Supplementary Fig 9). Instead, the largest subset (66.8%) had substrate and nucleotide simultaneously bound in an inward-facing conformation, with the NBDs partially reopened rather than fully dissociated as in the apo form (Fig. 2d). In this state, a density corresponding to ADP·VO₄ was clearly visible in one NBD but not in the other (Supplementary Fig. 15a, b), suggesting that ADP and phosphate are released in succession from one site then the other; alternatively this density could be an unresolved density due to the intrinsic flexibility of NBDs. The structures with these features are probably late post-hydrolytic intermediates or structures at an early stage of the inward-facing reset in which (at least one) ADP·VO₄ remains bound to a nucleotide-binding site while substrate rapidly rebinds within the central cavity, possibly as a result of the high substrate concentration used during grid preparation.

The GSH maps of both structures were well-resolved, enabling us to build precise atomic models. However, the density for CoPP was ambiguous, pointing to the absence of a single stable conformation. To improve the local resolution at the substrate-binding site, we employed 3D variability analysis followed by clustering (Supplementary Fig 6)^48^, or 3D classification without alignment using a TMD-focused mask (Supplementary Fig 9). This approach markedly improved the EM density of CoPP, revealing the propionate groups oriented toward the positively-charged cavity entrance and hydrophobic vinyl groups directed toward the apex of the cavity (Fig. 3a). Because the CoPP:GSH/ADP·VO₄-bound structure provided a more reliable and suitable density for the substrate, we used this structure to elucidate the substrate recognition mechanism of ABCB7.

Our data demonstrate that ABCB7 accommodates two stacked CoPP molecules within the central cavity, in a nearly vertical orientation relative to the membrane (Fig. 3a). In this configuration the cobalt ion of each CoPP bonds directly with the cysteine thiol groups of two GSH molecules. This binding mode is consistent with the observation that CoPP fails to stimulate ABCB7 activity in the presence of ophthalmic acid (Fig. 1b). We initially anticipated that ABCB7 would employ a substrate-binding mechanism similar to that of ABCB6, in which a single porphyrin molecule is coordinated between two GSH molecules^40^. However, structural comparisons revealed that, in the presence of substrate, there is greater separation between the NBDs of ABCB7 than between those of ABCB6 (26 Å vs 19 Å), resulting in a substrate-binding pocket approximately 10% larger in volume in the former (2,100 Å³ vs 1,900 Å³) (Fig. 3b)^47^. Consistent with this observation, the distance between the thiol groups of the two GSH cysteines is 9.7 Å in ABCB7, but only 6.5 Å in ABCB6 (Fig. 3c). The increased inter-thiol spacing in ABCB7 likely provides sufficient room to accommodate the second CoPP molecule within the central cavity. ABCB7 also provides direct contacts with the substrate – the conserved F426 aromatic pair form hydrophobic and van der Waals interactions with the bound CoPP (Fig. 3a).

Unlike the cysteine residue of GSH, the N-terminal glycine and the C-terminal glutamic acid of GSH are not directly involved in CoPP coordination. Instead, as in the binding of GSHs to ABCB6^40^, these termini interact with polar residues (N378 and Q381 on TM5) and basic arginine residues (R315 and R319 on TM4, and R432 on TM6), respectively, all of which are well conserved across species (Fig. 3a, d). This suggests that GSH is the primary docking site for metalloporphyrins and also stabilizes ABCB7 by filling the cavity and linking the transmembrane helices. Our structural findings align with the results of site-specific mutagenesis (Fig. 3e, f): alanine substitutions at residues R315, R319, N378, Q381, F426, and R432 lead to partial or complete loss of substrate-stimulated ATPase activity. Interestingly, loss of positive charge through replacement of the three arginine residues, either individually or in combination (R315A, R319A, and R432A), seems to disrupt the coupling between substrate binding and ATP hydrolysis, leading to a futile state in which the protein wastefully hydrolyzes ATP without normal conformational switching. As a result, basal ATPase activity is abnormally elevated and there is complete loss of substrate stimulation (Fig. 3f).

We also collected cryo-EM data for ABCB7 in complex with hemin:GSH (Supplementary Fig 16). Although the hemin:GSH-bound reconstruction reached 3.1 Å resolution, the hemin density appeared somewhat smeared, reflecting conformational heterogeneity and/or reduced occupancy. This observation is also consistent with the weaker ATPase stimulation by hemin compared with CoPP, as the greater structural flexibility associated with hemin may lead to less efficient priming of the transporter for ATP hydrolysis than in the case of the more stably engaged CoPP. Nevertheless, the hemin:GSH map supports a nearly identical binding mode: two hemin molecules occupy the same TM cavity in a stacked arrangement, and the associated GSH adopts a similar position, engaging the same set of key interacting residues. Together, these data support a conserved binding mode for hemin and CoPP in ABCB7.

### CoPP partitioning into the membrane

**Fig. 4:**
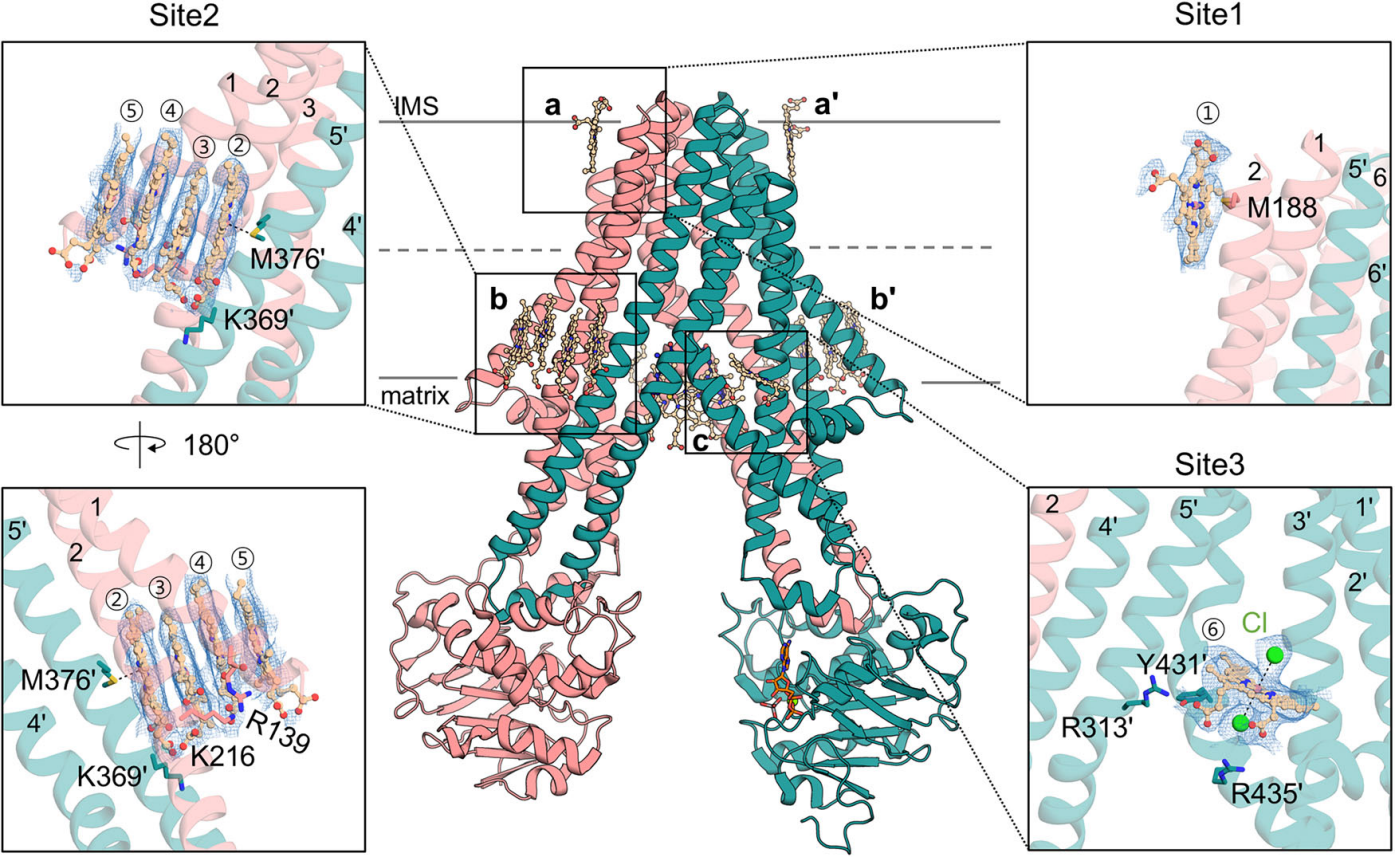
ABCB7-mediated recruitment of substrate within the membrane. **a–c** The external substrate-binding sites identified in the lipid bilayer. **a** membrane outer leaflet, **b** inner leaflet, and **c** lateral gate. Key interacting residues and coordinating ions were analyzed using LigPlot+ and are shown as sticks and spheres, respectively, in the close-up views.

In the substrate-bound structure we observed an unexpected intercalation of CoPP molecules within the membrane bilayer (Fig. 4a–c). At site 1, one CoPP per ABCB7 monomer adopted a perpendicular orientation relative to the membrane plane, with its two propionic acid groups directed toward the mitochondrial intermembrane space (Fig. 4a). This CoPP lies adjacent to TM 2, where residue M188 directly coordinates its central Co atom. At site 2, four CoPP molecules adopt a stacked arrangement, with residue M376 on TM5 serving as the axial ligand for the proximal CoPP closest to the transporter (Fig. 4b). These CoPPs are rotated 180° relative to the CoPP at site 1. In this orientation, their hydrophobic side chains of CoPP insert into the membrane core, whereas the propionate groups project toward the phospholipid head groups of the inner membrane, forming electrostatic interactions with basic residues R139, K216, and K369. At site 3 (also known as the lateral gate), one CoPP molecule is oriented almost parallel to the membrane plane (Fig. 4c). Unlike at sites 1 and 2, where methionine residues provide axial coordination for CoPP, binding at site 3 appears to be mediated by axial chloride ligands^49^, which are stabilized through interactions with R313, Y431, and R435. These structural observations are consistent with previous studies showing that heme spontaneously partitions into lipid bilayers and accumulates within the membrane^46,50,51^. Molecular dynamics (MD) simulations of the bacterial heme transporter CydDC have shown that membrane-embedded heme undergoes a ~ 90° rotation at lateral gate during entry into the transporter^52^. Furthermore, the simulations have demonstrated that heme can form stable dimeric and higher-order stacks via face-to-face porphyrin ring interactions^53^. Importantly, membrane-anchored FECH, which catalyzes the final step of heme biosynthesis, is known to bridge and physically interact with both ABCB7 and ABCB10^19,20^. These associations raise the intriguing possibility that newly synthesized heme generated by FECH may be directly handed off to ABCB7 through the lipid bilayer for subsequent export.

### The occluded structure of ABCB7 in the ATP-bound, pre-hydrolytic state

**Fig. 5:**
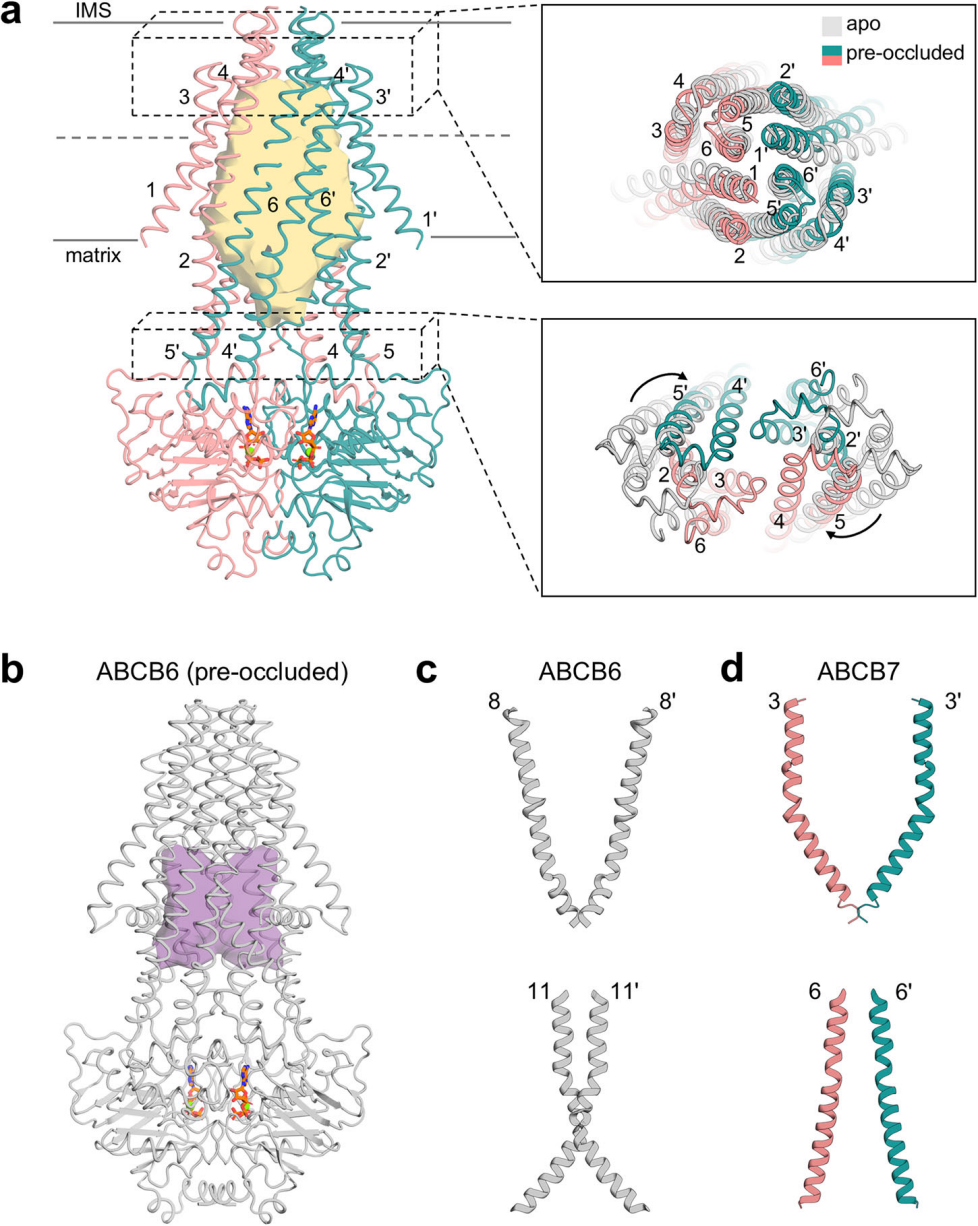
Cryo-EM structure of the ABCB7 E634Q mutant in the pre-hydrolytic occluded state. **a** Overall structure of the ABCB7 E634Q mutant in the occluded state. Substrate-binding cavities are colored yellow. Close-up views show structural rearrangements of the TM helices between the apo (grey) and pre-occluded states (salmon and teal). ATP is shown as sticks (orange) and Mg^2+^ as a sphere (green). **b** Cartoon representation of the human ABCB6 E752Q mutant in the pre-occluded state (PDB ID 7EKL). The substrate-binding cavity is colored purple. **c**,**d** Comparison of the TM helices responsible for the difference in cavity volume between human ABCB6 (**c**) and ABCB7 (**d**). The conformations of TM8 and TM11 in ABCB6, and the corresponding TM3 and TM6 in ABCB7, are shown as ribbon representation.

We captured the occluded conformation of human ABCB7 (E634Q mutant) in the presence of CoPP and Mg^2+^/ATP. The transition from the apo inward-fully open state to this occluded state involves distinct conformational changes in both the TMDs and NBDs. Within the TMDs, the upper segments are stabilized by tight helical packing, while the bottom halves of the TM helices undergo coordinated inward movements, sealing the cytoplasmic gate (Fig. 5a). This conformational change results in a continuous and enlarged internal cavity of ~3200 Å³. At the same time, the two NBDs form a canonical closed dimer with a head-to-tail fold (Supplementary Fig. 15c, d). This arrangement allows us to see the side-chain interactions that define ATP binding and hydrolysis mediated by the signature motif, Walker A and B elements, and the Q-loop.

A distinctive feature of the ATP-bound ABCB7 is its unusually large substrate-binding cavity, which is markedly expanded compared to that of ABCB6 (E752Q, ~220 Å³) in the same functional state (Fig. 5b). Structural superposition indicates that this enlargement primarily arises from the different conformations of TM3 and TM6 lining the transport pathway (Fig. 5c, d). In ABCB6, TM8 (corresponding to TM 3 in ABCB7) shifts inward, while TM11 (corresponding to TM6 in ABCB7) exhibits a kinked, tightly packed arrangement (Fig. 5c). By contrast, in ABCB7, TM3 undergoes an outward displacement and TM6 adopts a straightened conformation, together generating additional internal volume (Fig. 5d). These differences suggest that the two transporters represent distinct functional states, despite both structures being determined using catalytically inactive E-to-Q mutants in the presence of ATP. ABCB7 exhibits an early pre-hydrolytic state with a sufficiently large cavity to accommodate the substrate prior to transitioning to an outward-facing release conformation. In contrast, ABCB6 corresponds to a late pre-hydrolytic state, in which collapse of the central cavity pushes the substrate to be expelled and prevents its rebinding to the transporter.

### Homology modelling of the outward-facing conformation of ABCB7

Despite extensive efforts, we were unable to capture the outward-facing conformation of ABCB7, suggesting that this state is very short-lived so as to prevent substrate re-entry after release^54^. To gain mechanistic insight into the complete transport cycle of ABCB7, we constructed a homology model of its outward-facing conformation using MODELLER^55,56^. Given the high sequence identity and functional conservation with ABCB6 (Supplementary Fig 1), we selected the outward-facing structure of its W546A mutant stabilized with coproporphyrinogen III and ADP·VO₄ (PDB ID 8K7C) as the template^57^. The resulting model enabled accurate alignment of conserved residues critical for substrate recognition and transport, indicating that it represents a functionally plausible outward-facing conformation of ABCB7.

**Fig. 6:**
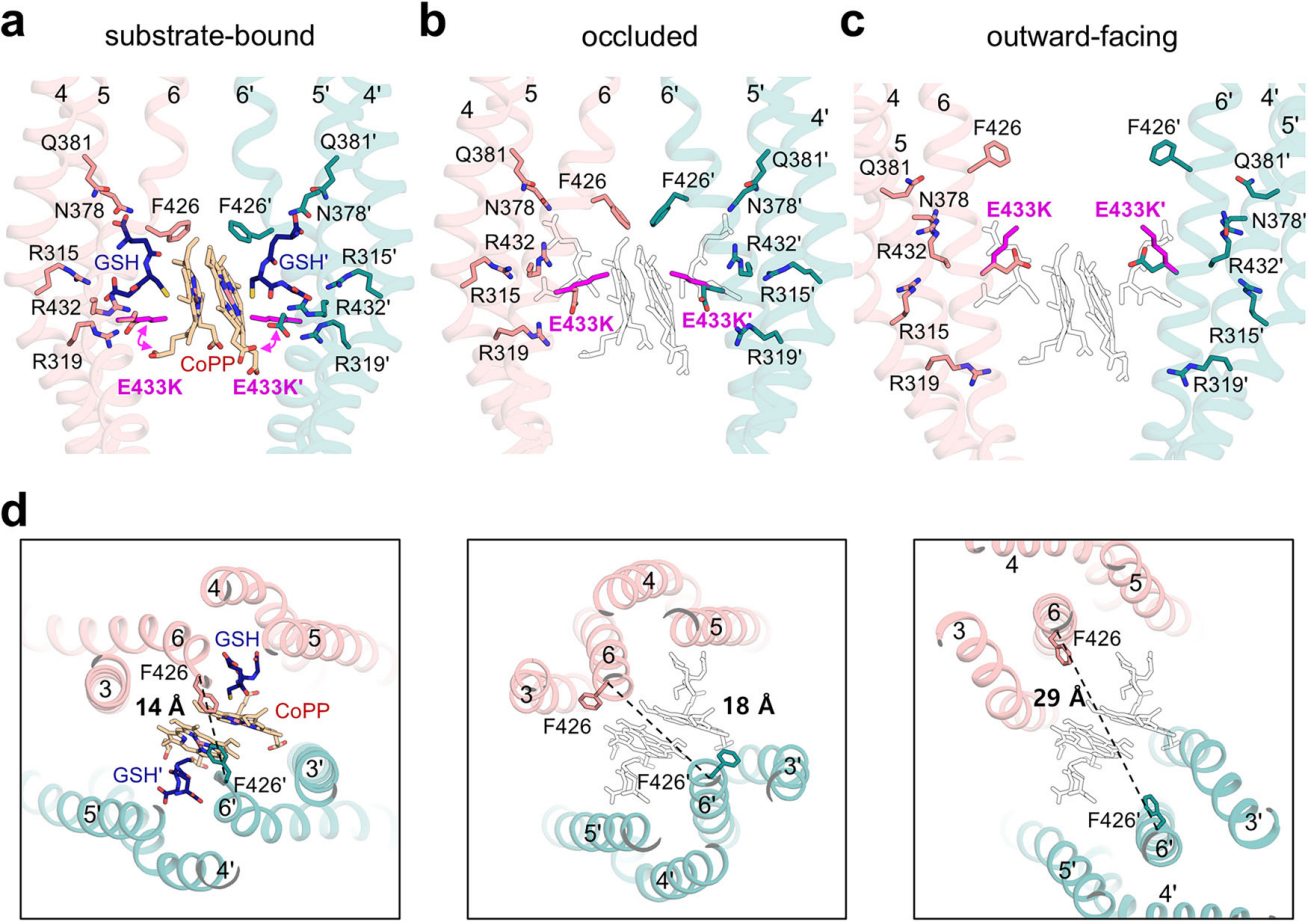
Homology model of human ABCB7 in the outward-facing conformation. **a–c** Possible mechanism of substrate efflux. Close-up views show the substrate-binding sites of each conformation viewed within the plane of the membrane. Bound substrate and key interacting residues are shown as sticks. CoPP:GSH complexes, superimposed on the pre-occluded and outward-facing models, are shown as empty black sticks. The XLSA/A-associated missense mutation (E433K) is highlighted in magenta. **d** Top views corresponding to (**a–c**).

This homology-based structural model yields several important insights. First, it has the hallmark features of the outward-facing conformation of ABC transporters, including a closed NBD dimer and an open exit pathway from mitochondrial matrix toward the intermembrane space for directional release (Supplementary Fig 17). Second, it allows one to visualize the substrate transport mechanism of ABCB7. Compared with the substrate-bound state, the occluded conformation exhibits modest but apparent backbone displacements near the central cavity, resulting in key substrate-interacting residues shifting slightly away from the bound substrate. In this state, the side chain of Q381 shows the largest movement, increasing its distance from the glycine moiety of GSH from 3.7 Å to 5.4 Å (Fig. 6a, b). These rearrangements probably reduce the affinity of ABCB7 for CoPP, so priming the transporter for substrate release. By contrast, in the outward-facing state the TM backbone undergoes substantial rearrangements (Fig. 6c). In consequence, residues lining the central cavity shift away from the bound substrate and the upper cavity expands to facilitate its release. Furthermore, the conserved gating residue F426 swings outward in a stepwise manner from closed to partially-open to fully-open to promote substrate release (Fig. 6d). This motion can be seen by the increase in the inter-subunit F426–F426’ distance from 14 Å (substrate-bound) to 18 Å (occluded) and then to 29 Å (outward-facing). Third, human ABCB7 has been implicated in X-linked sideroblastic anemia with ataxia (XLSA/A), where four missense mutations (E208D, I400M, V411L, and E433K) have been identified in patients^14,22–24^. Previous structural analysis has shown that three of these residues (E208, I400, and V411) lie within the dimer interface in the inward-facing state, suggesting that the corresponding mutations impair dimer assembly and/or conformational transitions required for substrate transport^35^. By contrast, E433 does not contribute to the dimer interface, and the functional consequence of this mutation was previously unexplained when using the inward-facing model. Our experimental structures and homology model now provide a mechanistic rationale: residue E433 is exposed near the substrate-binding pocket (Fig. 6a) or along the exit pathway (Fig. 6b, c). Hence, substitution of glutamate with the longer, positively charged lysine probably causes steric hindrance that disrupts stable substrate binding and/or slows substrate release due to enhanced electrostatic interactions between the transporter and the polar side chains of porphyrin substrates.

## Discussion

CoPP is a non-natural compound that shares key chemical properties with heme and exerts various physiological effects^58^. Specifically, it has been shown to potently induce the expression of endoplasmic reticulum (ER)-anchored heme oxygenase-1 (HO-1)^59^. HO-1 enzyme catalyzes the degradation of cytotoxic free heme into ferrous ions (Fe²⁺), carbon monoxide (CO), and biliverdin, which is subsequently converted to bilirubin by biliverdin reductase^60,61^. HO-1 activation has significant cytoprotective effects, including antioxidant^62^, anti-inflammatory^63^, and anti-apoptotic actions^64^. As CoPP structurally mimics heme but cannot be efficiently degraded by HO-1, CoPP treatment has been proposed as a potential therapeutic strategy for oxidative stress-related diseases, including cardiovascular disease^65,66^, diabetes^67^, obesity^68^, septic insults^65^, and metabolic syndrome^69^. In this study we have shown that ABCB7 selectively couples ATP hydrolysis to transport of hemin and CoPP, and that this process is strictly GSH-dependent (Fig. 1). Expanding on the established functions of ABCB7 in maintaining mitochondrial iron and redox homeostasis, these results suggest novel roles for ABCB7 in heme export^19,20^ and the HO-1-dependent cytoprotective pathway^62–64,70–73^.

It is noteworthy that the apparent K_m_ for CoPP determined in vitro was higher than the low nanomolar concentrations reported for the labile heme pool in vivo (Fig. 1b)^74^. One plausible explanation is that ABCB7 accesses transiently elevated local substrate concentrations through spatial coupling to FECH^19,20^. Indeed, ABCB7 has been reported to associate with a complex containing FECH, mitoferrin-1, ABCB10, and additional partners^75^. Such organization could promote substrate channeling, enabling efficient substrate handoff between components of the complex without release into the bulk aqueous phase and thereby increase local substrate availability near ABCB7. Future work will establish whether incorporation of ABCB7 into such assemblies alters its apparent substrate affinity and turnover.

Our structural and functional characterizations offer significant insights into the substrate recognition and transport mechanism of ABCB7. Unlike ABCB6, which accommodates only one porphyrin coordinated by two GSHs, the unusually large cavity of ABCB7 has the capacity to accommodate two CoPP:GSH molecules concurrently (Fig. 3a). We hypothesize that this dual-cargo system increases export efficiency to meet high demands during upregulated FECH-driven heme production. Because free CoPP is highly hydrophobic and partitions strongly into the lipid bilayer (Fig. 4), the formation of a CoPP:GSH complex is expected to introduce hydrophilic functionalities to CoPP. This modification likely stabilizes CoPP once it enters the ABCB7 cavity as this is primarily lined with polar and charged residues (Fig. 3a). Thus, GSH may serve as a cofactor that facilitates loading and stabilization of the substrate in the transporter cavity. Under oxidative stress, the cellular GSH pool can become severely limited. However, mitochondrial GSH homeostasis is preferentially maintained by NADPH-dependent regeneration^76^ and import pathways^77^. Consequently, we propose that ABCB7-mediated porphyrin transport might be attenuated under such conditions but would not necessarily be abolished.

In this study we used a high CoPP concentration (1 mM) during grid preparation to maximize substrate occupancy and obtain well-defined densities in cryo-EM reconstructions. At 10-fold lower CoPP concentration (100 µM), the stacked CoPP density within the cavity was essentially unchanged, indicating that the accommodation of stacked porphyrins is a robust structural feature of ABCB7 rather than an artifact of excess substrate (Supplementary Fig 18). We note, however, that the reported concentration of free heme in the mitochondrial matrix is in the low nanomolar range^74^. At such physiological concentrations, ABCB7 may also engage a single heme species rather than a stacked configuration. However, formation of this low occupancy state would likely require substantial inward movement of the TMDs to reduce and compact the cavity volume. Such a large, energetically demanding rearrangement may therefore occur infrequently and/or exhibit a lower transport rate.

Our structures also revealed the presence of unexpected external substrate-binding sites within the lipid phase (Fig. 4). In particular, in the presence of 1 mM CoPP, four CoPP molecules assemble into a stacked array at the level of the membrane inner leaflet (site 2). However, at a low CoPP concentration the membrane-partitioned CoPP density was no longer detectable. This suggests that excess porphyrins partition strongly into the lipid bilayer where the hydrophobic environment stabilizes the porphyrin tetrapyrrole ring and limits the aggregation or redox reactivity that is prone to occur with free porphyrins in aqueous solution^78,79^. Interestingly, we did not observe comparable membrane-partitioned densities when grids were prepared with 1 mM hemin. A plausible explanation is that hemin exists as a more chemically and conformationally heterogeneous ensemble: its axial chloride ligand can be readily exchanged for water/hydroxide, and the variability in central iron coordination and redox chemistry may hinder the formation of a stable, membrane-associated stacked hemin population^80^. By contrast, Co(III)-porphyrins typically exhibit more inert axial ligand coordination than Fe(III)-porphyrins^49^. Accordingly, CoPP may adopt a more uniform configuration, enabling it to survive during 3D averaging in cryo-EM data processing. Another possibility is that hemin may be more effectively buffered by GSH in solution, reducing its membrane intercalation and further lowering any ordered density in the membrane.

A recent cryo-EM study of another porphyrin transporter, human ABCB10, demonstrated that despite sharing the overall fold with ABCB7, the two transporters have diverged markedly in substrate recognition^42^. The structures supported a role for ABCB10 as a biliverdin exporter and revealed a predominantly hydrophobic substrate-binding pocket with only limited positively charged patches. In addition, whereas ABCB7 uses GSH as a cofactor for substrate recognition, ABCB10 appears to engage cholesterol, further suggesting a substrate recognition mode distinct from that observed for ABCB7. Consistent with these structural features, sequence alignment indicates that the key residues forming the ABCB7-CoPP interaction network are not well conserved in ABCB10 (Fig. 3d). Collectively, the available structural and biochemical evidence indicates that ABCB10 is a parallel component of mitochondrial heme metabolism, rather than a transporter sharing its substrate-binding mechanism with ABCB6 or ABCB7.

**Fig. 7:**
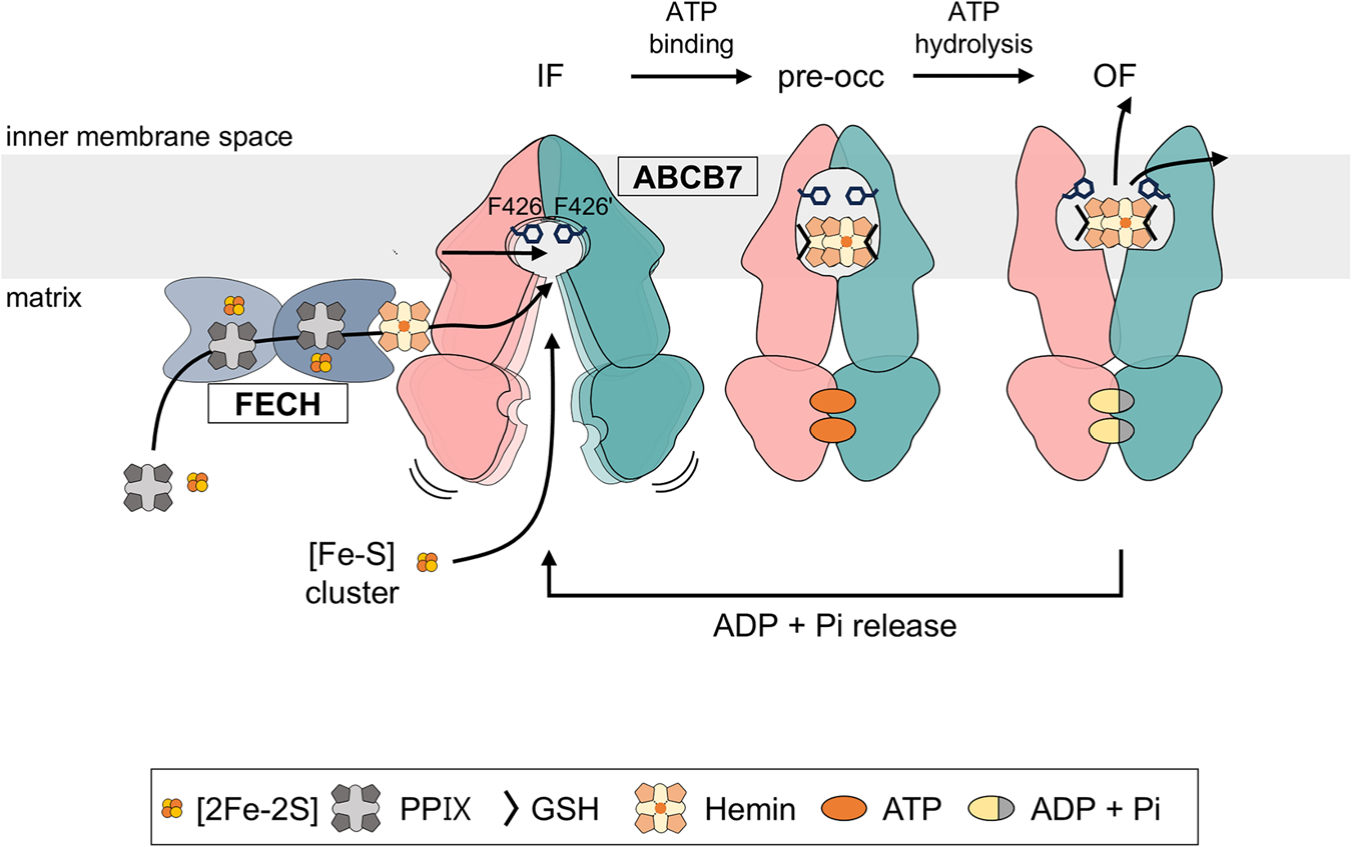
Proposed mechanism of substrate transport by ABCB7. ABCB7 exhibits intrinsic conformational dynamics, transitioning between multiple inward-facing states. It is able to associate with both soluble Fe-S cluster and lipophilic heme synthesized by FECH. In this conformation, the F426 gate remains closed. ATP and substrate binding induce a conformational transition to an occluded state, in which the two NBDs move closer together and the TMDs trap the substrate inside the sealed central cavity. ATP hydrolysis triggers transient opening of the F426 gate, allowing substrate release toward the mitochondrial intermembrane space. Finally, dissociation of ADP and phosphate, either sequentially or simultaneous, resets ABCB7 to its inward-facing conformation. For clarity, only FECH is shown in the model as a possible interacting partner of ABCB7.

The canonical transport mechanism of many ABC transporters follows a membrane-mediated “vacuum cleaner” paradigm in which hydrophobic compounds accumulate within the lipid bilayer and are funneled laterally into the translocation pathway of the transporter^81–84^. However, such a process would be inefficient and potentially detrimental for heme handling, because heme is a potent pro-oxidant that can disrupt lipid organization and promote lipid peroxidation^85,86^. Since ABCB7 has been proposed to participate in multi-protein assemblies containing heme biosynthetic enzymes, transport/accessory factors, and chaperone-like components^75^, we therefore propose a protein-mediated substrate handoff model in which ABCB7 engages heme through direct or indirect interactions with FECH and other partner proteins. In this model, heme may undergo transient interactions at the matrix-facing surface of ABCB7 prior to entry into the cavity, thereby minimizing the residence time of free heme within the membrane and enabling vectorial export from mitochondria to the cytosol (Fig. 7). In such a framework, conserved residue F426 functions as a gatekeeper that regulates substrate trapping and release during the transport cycle. Finally, our structural insights provide a mechanistic basis for the role of the E433K variant in XLSA/A disease (Fig. 6)^14,21^. Substitution of E433 with lysine likely disrupts substrate coordination and release through steric hindrance and unfavorable electrostatic interactions, respectively. These structural insights may pave the way for therapeutic strategies aimed at restoring ABCB7 function in disease contexts.

## Methods

### Cloning, expression, and purification

Human ABCB7 was produced and purified using a previously described protocol, with slight adjustments^35^. Briefly, the N-terminal truncated form of *Homo sapiens* ABCB7 (residues 71–752) was codon-optimized and synthesized by Gene Universal. It was cloned into the pVL1393 baculovirus transfer vector (BD Biosciences) with a C-terminal thrombin-cleavable enhanced green fluorescent protein (eGFP) tag and a decahistidine (10×His) affinity tag. Site-directed mutagenesis was performed by overlap extension polymerase chain reaction (PCR). For protein expression, recombinant ABCB7 plasmids were transfected into *Spodoptera frugiperda* (Sf9) cells using Cellfectin™ II transfection reagent (Gibco) along with BestBac 2.0 linearized baculovirus DNA (Expression Systems). Amplified baculovirus stocks were used to infect High Five (Hi5) cells (Expression Systems). Fluorescence microscopy (DM IL LED, Leica Microsystems) was used to monitor protein expression in infected Hi5 cells, which were subsequently harvested 72 h post-infection.

All purification steps were conducted on ice or at 4 °C. Harvested cells were resuspended in lysis buffer containing 50 mM MES-NaOH pH 6.5, 200 mM NaCl, 10 mM MgCl₂, 10% glycerol, 1 mM phenylmethylsulfonyl fluoride (PMSF), and 40 μg/mL DNase I. The resuspended cells were lysed by sonication using a Branson sonifier (3.2 mm tip) at 45% amplitude, with 10 s on/off pulses, for a total of 3 min. The membrane fraction was isolated via ultracentrifugation at 300,000 × g for 90 min and resuspended in lysis buffer. Membranes were solubilized in 1% (w/v) lauryl maltose neopentyl glycol (LMNG, Anatrace) and 0.1% (w/v) cholesteryl hemisuccinate (CHS, Anatrace) by gentle mixing overnight. Insoluble debris was removed by ultracentrifugation at 300,000 × g for 1 h. The solubilized protein was purified using an anti-GFP-DARPin-conjugated NHS agarose affinity resin (Cytiva)^87^. The resin was washed extensively with a buffer containing 30 mM MES-NaOH pH 6.5, 200 mM NaCl, 5% (v/v) glycerol, 0.1% (w/v) LMNG, and 0.01% (w/v) CHS. A second wash was performed using a buffer with a lower detergent concentration [0.02% (w/v) LMNG and 0.002% (w/v) CHS]. The protein was eluted from the resin via on-column thrombin cleavage (Lee Biosolutions) in a buffer containing 30 mM MES-NaOH pH 6.5, 200 mM NaCl, 5% glycerol, 0.02% (w/v) LMNG, and 0.002% (w/v) CHS. Further purification was performed by size-exclusion chromatography on a Superdex 200 Increase 10/300 GL column (Cytiva) equilibrated with a buffer containing 25 mM MES-NaOH pH 6.5, 150 mM NaCl, 0.01% (w/v) LMNG, and 0.001% (w/v) CHS. Peak fractions were analyzed by SDS-PAGE, and the purified protein was concentrated to ~10 mg/mL with Amicon ultrafiltration concentrators (Merck Millipore, 100 kDa cut-off).

### ATP hydrolysis assay

The ATP hydrolysis activities of wild-type ABCB7 and its mutants were measured by the NADH-coupled method^88^ or the molybdate assay^89^ as previously described. Briefly, in the NADH-coupled assay, 14 μg (1.2 μM) of detergent-purified protein was added to 150 μL of reaction buffer containing 50 mM HEPES-KOH pH 8.0, 60 μg/mL pyruvate kinase (Roche), 16 mg/mL lactate dehydrogenase (Roche), 4 mM phosphoenolpyruvate (Roche), 0.3 mM NADH, 3 mM ATP, 10 mM MgCl₂, 0.01% (w/v) LMNG, and 0.001% (w/v) CHS. Reactions were performed in the presence or absence of substrate as indicated. They were monitored at 37°C by recording the decrease in NADH absorbance at 340 nm (ε = 6220 cm⁻¹ M⁻¹) every 10 s for 3 min. Basal ATPase activity was assessed using an ATP gradient up to 6 mM.

In the molybdate assay, 12 μg (1.6 μM) of purified protein was incubated with 50 μL of reaction buffer containing 50 mM HEPES-KOH (pH 7.0), 70 mM KCl, 10 mM MgCl₂, 0.01% (w/v) LMNG, and 0.001% (w/v) CHS. Various porphyrins (40 μM) or thiol compounds (5 mM) were added, either alone or in combination with 1 mM GSH, and samples were incubated on ice for 30 min. ATP hydrolysis was initiated by adding 3 mM ATP, followed by incubation at 37 °C for 30 min. The reaction was then quenched by adding 50 μL of 10% (w/v) sodium dodecyl sulfate (SDS). Subsequently, 100 μL of a detection solution containing 5 mM ammonium molybdate, 2 mM zinc acetate, and 5.5% (w/v) ascorbic acid was added. The reaction mixture was incubated in the dark at room temperature for 20 min, and the concentration of released inorganic phosphate (Pi) was measured by absorbance at 850 nm with a microplate absorbance reader (FlexStation 3, Molecular Devices). ATP hydrolysis rates were determined using a potassium phosphate standard curve. Curve fitting to the Michaelis–Menten equation via nonlinear regression was performed using PRISM 10.0 (GraphPad Software).

### Nanodisc reconstitution

A truncated variant of human apolipoprotein A-I (ApoA1; residues D44–L243) lacking the N-terminal 43 residues was prepared as previously described^90^. Briefly, ApoA1 was cloned into the pET21a vector (Novagen) with a C-terminal hexahistidine (6×His) tag and a thrombin cleavage site. The construct was expressed in *Escherichia coli* BL21(DE3) cells grown in Luria Bertani medium at 37 °C. Protein expression was induced with 1 mM isopropyl-β-D-thiogalactopyranoside (IPTG; GoldBio) at an OD₆₀₀ of 0.6–0.7, followed by incubation at 30 °C for 5 h. Cells were harvested, resuspended in lysis buffer containing 20 mM Tris-HCl pH 8.0, 200 mM NaCl, 10 μg/mL DNase I, and 0.1 mM PMSF, and disrupted by sonication. After centrifugation at 40,000 × g for 1 h, the supernatant was applied to Ni–NTA resin equilibrated with lysis buffer. Bound proteins were washed with lysis buffer supplemented with 30 mM imidazole and eluted with 300 mM imidazole. The 6×His tag was removed by thrombin digestion overnight at 4 °C, and ApoA1 was further purified by anion exchange chromatography (HiTrap Q, Cytiva) followed by size-exclusion chromatography (Superdex 200 Increase 10/300 GL, Cytiva).

For nanodisc reconstitution, *E. coli* polar lipid extract (Avanti Polar Lipids) dissolved in chloroform was dried under nitrogen to form a lipid film, which was rehydrated in 25 mM MES-NaOH pH 6.5, 150 mM NaCl, and 0.0174% (w/v) n-dodecyl-β-D-maltopyranoside (DDM, Anatrace). The lipid suspension was incubated at 37 °C for 30 min and briefly sonicated to disperse aggregates. Solubilized lipids were mixed with ApoA1 and purified ABCB7 at molar ratios of 150:4:1, and incubated at 4 °C for 2 h. Detergent was removed by three successive additions of Bio-Beads SM-2 (Bio-Rad). Empty nanodiscs and excess ApoA1 were removed by size-exclusion chromatography equilibrated in 25 mM MES-NaOH pH 6.5 and 150 mM NaCl. Fractions containing homogeneous nanodisc-reconstituted ABCB7 were collected for preparing cryo-EM grids and ATPase assays.

### Grid preparation and data collection

To prepare grids, 3.5 µL of detergent-purified ABCB7 (5 mg/mL) or nanodisc-reconstituted protein (1.5 mg/mL) was applied to glow-discharged (15 mA, 90 s) Quantifoil 1.2/1.3 300 mesh Au holey carbon grids. The grids were blotted for 4.5–5 s with a blot force of 3–5 at 4 °C and 100% humidity, followed by plunge-freezing in liquid ethane using a Vitrobot Mark IV (Thermo Fisher Scientific). For the substrate-bound state, detergent-purified wild-type ABCB7 was incubated with 1 mM CoPP and 1 mM GSH for 10 min at 37 °C prior to vitrification. For the ATP-bound state, the detergent-purified E634Q mutant was incubated with 1 mM CoPP, 1 mM GSH, 3 mM ATP, and 3 mM MgCl₂ for 10 min at 37 °C before plunge-freezing. Nanodisc samples were incubated with 0.5 mM CoPP, 0.5 mM GSH (or 0.1 mM CoPP, 0.1 mM GSH), 3 mM ATP, 3 mM MgCl₂, and 3 mM sodium orthovanadate (Na₃VO₄) at 22 °C for 30 min prior to vitrification.

Cryo-EM data for detergent-purified samples were collected with a 300 kV Titan Krios G4 transmission electron microscope (Thermo Fisher Scientific) equipped with a K3 camera and BioQuantum energy filter (slit width 20 eV). Data were acquired with EPU software (Thermo Fisher Scientific) in counting mode at ×130,000 magnification with a calibrated pixel size of 0.673 Å. Movies were recorded with an exposure time of 1.49 s and dose-fractionated into 50 frames with a total dose of 50 e^−^/Å^2^. The electron dose rate was 15 e^−^/pix/sec (1 e^−^/Å^2^/frame), and the defocus range was set between -0.8 and −2.2 μm with 0.2 μm steps. Cryo-EM data for nanodisc samples were collected on a 300 kV Titan Krios G4 transmission electron microscope equipped with a cold field emission gun (CFEG) and a Falcon 4i direct electron detector (Thermo Fisher Scientific). Movies were recorded in counting mode at a nominal magnification of ×165,000, corresponding to a calibrated pixel size of 0.76 Å. Exposures lasted 2.95 s each and were recorded as a movie of 40 frames with a total accumulated electron dose of 50 e^−^/Å^2^.

### Cryo-EM data processing

Data for the apo and substrate-bound conformations were processed using a RELION v4.0^91,92^ and cryoSPARC v4.3.1^93^. Briefly, the beam-induced motion of movie stacks was corrected with MotionCor2^94^, and the contrast transfer function (CTF) was estimated using CTFFIND4^95^. Low quality micrographs were removed, and particles were auto-picked via the Laplacian-of-Gaussian method using a subset of images. Picked particles were extracted and subjected to initial 2D classification. Particles from high-quality 2D classes were used for template-based particle picking across entire micrographs. The extracted particles were then imported into cryoSPARC and used to generate ab initio models. Next, 3D heterogeneous refinement was performed for each model with C1 symmetry, and the best class was selected for 3D homogeneous refinement. The particles were then imported back into RELION for Bayesian polishing^96^. Polished particles were re-imported into cryoSPARC for a second round of ab initio modeling and 3D heterogeneous refinement. For the apo state, a final set of 216,118 particles was subjected to 3D homogeneous refinement, followed by local CTF refinement. The final map of the apo structure was refined to 3.0 Å using C2 symmetry and sharpened with a calculated B-factor of 116.2 Å^2^. For the substrate-bound conformation, 3D Variability Analysis (3DVA, *n* = 10), followed by 3D Variability Display, was performed to improve the resolution at the substrate-binding site^48^. The best clusters contained 165,153 particles and underwent final 3D homogeneous refinement with C1 and C2 symmetry. Subsequent non-uniform refinement yielded maps at 3.4 Å resolution, with overall B-factors of 144.7 Å² and 162.2 Å², respectively^97^.

For the ATP-bound conformation, 7542 micrographs were imported and processed in CryoSPARC. The movies were motion-corrected, and CTF values were calculated using patch CTF estimation. Blob picking followed by template-based picking were used to select ~2,400,000 particles that were sorted by three rounds of 2D classification. The resultant ~800,000 particles were subjected to multiple rounds of ab initio reconstruction and 3D heterogeneous refinement. Two classes were obtained at 3.8 Å resolution, accounting for 51% and 25% of the total particles, respectively. Homogeneous refinement of these models resulted in a merged 2.6 Å resolution map. Further processing, including local motion correction and CTF refinements, improved the map quality. Final non-uniform refinement using C1 and C2 symmetry yielded resolutions of 2.5 Å and 2.3 Å, with B-factors of 83.5 Å^2^ and 83.3 Å^2^, respectively.

For the substrate- and ADP·VO_4_-bound sample, a data-processing workflow similar to that used for the ATP-bound conformation was employed, with an additional focused 3D classification step. Focused classification using a TMD mask was used to isolate substrate-bound particles and remove low-quality subsets. The selected particles were subsequently refined by 3D homogeneous and local refinement, yielding a final map at 2.8 Å resolution with a B-factor of 83.9 Å² (C1 symmetry). Details of the data processing workflow are presented in Supplementary Figs 3–14. Overall resolution was estimated using a gold-standard Fourier Shell Correlation (FSC) cut-off of 0.143 between the two independently refined half-maps^98^. Local resolution was calculated from the two half-maps in cryoSPARC^93^ and visualized in UCSF Chimera^99^.

### Model building and refinement

The previously-reported cryo-EM structure of human ABCB7 (PDB ID 7VGF)^35^ was used as the initial model for the TMD and NBD regions of the inward-facing conformations. For the occluded conformation, the structure of human ABCB10 in the corresponding state, determined in our laboratory but not yet published, served as the initial model. The individual domains were fitted into the final maps using UCSF Chimera^99^ and refined using the rigid body and morphing parameters in the real-space refinement tool of PHENIX suite^100^. Residue assignment and rotamer coordinates were initially refined in Coot^101^. Model quality was further improved by multiple cycles of real-space refinement in PHENIX and manual model building in Coot. Amino acids in regions with poor density were modeled as poly-alanine. The final structures were validated using MolProbity^102^ and the PDB validation server (https://validate-rcsb-2.wwpdb.org/). Residues with poor rotamer conformations and Ramachandran outliers were corrected in Coot. Refinement and validation statistics are summarized in Supplementary Table 1. All structural figures were generated using UCSF Chimera^99^, Chimera X^103^, and PyMOL (Schrödinger, LLC, https://pymol.org/2/).

### Homology modeling of outward-facing ABCB7

The outward-facing conformation of human ABCB7 was modeled using the structure of human ABCB6 in the outward-open state (PDB ID 8K7C) as reference template^57^. A monomeric model of ABCB7 was first generated from chain A of ABCB6 using the automodel class in MODELLER (v10.6)^55,56^. The top-ranked model, selected on discrete optimized protein energy (DOPE) scores^104^, was duplicated and arranged according to the C2 symmetry observed in the occluded ABCB7 structure to generate a full homodimeric assembly. The resulting model was validated using MolProbity^102^, ensuring optimal backbone geometry and side-chain rotamers.

### Statistical analysis

Experimental data are presented as mean ± standard error of the mean (SEM). Statistical analyses and graphical visualizations were performed using GraphPad Prism version 10.0 software (GraphPad Software). Statistical differences were assessed by two-way ANOVA with post hoc multiple comparisons: Tukey’s test for Fig. 1b and Dunnett’s test for Figs. 1d, 3e, f. Statistical significance is denoted by asterisks in Figs. 1b and 3f, where **** *P* < 0.0001.

### Reporting summary

Further information on research design is available in the Nature Portfolio Reporting Summary linked to this article.

## Supplementary information

Supplementary Information

Description of Additional Supplementary Materials

Supplementary Data 1

Reporting Summary

Transparent Peer Review file

## Data Availability

The atomic coordinates have been deposited in the Protein Data Bank under accession codes 23PH (apo), 23PG (substrate and ADP·VO_4_-bound) and 23PI (ATP-bound). The cryo-EM density maps are available in the Electron Microscopy Data Bank with accession codes EMD-69144 (apo), EMD-69143 (substrate and ADP·VO_4_-bound) and EMD-69145 (ATP-bound). Source data are provided with this paper and can be found in Supplementary Data 1.
