# Supplementary material for: Cryo-EM structures of human ABCB7 reveal the molecular basis of mitochondrial matrix heme export
Source: Commun Biol. 2026 May 9;9:970. doi: 10.1038/s42003-026-10223-x (PMC13376393; doi:10.1038/s42003-026-10223-x)
Supplement: Supplementary file 1 — Supplementary Information [file 42003_2026_10223_MOESM1_ESM.pdf]

# Supplementary Information

for

## Cryo-EM structures of human ABCB7 reveal the molecular basis of mitochondrial matrix heme export

Seulgi Ju[1], Seung Hun Choi[1], Hyeon You Lee[1] & Mi Sun Jin[1,2,*]

[1]School of Life Sciences, Gwangju Institute of Science and Technology (GIST), Gwangju, Republic of Korea

[2]Integrated Institute of Biomedical Research, Gwangju Institute of Science and Technology, Gwangju 61005, Republic of Korea

*To whom correspondence should be addressed. Email: misunjin@gist.ac.kr (M.S.J.)

```
hABCB7
hABCB6      ------------------------------MVTVGNYCEAEGPVGPAWMQDGLSPCFFFT   30
hABCB10

hABCB7      ------------------------------------------------------------
hABCB6      LVPSTRMALGTLALVLALPCRRRERPAGADSLSWGAGPRISPYVLQLLLATLQAALPLAG   90
hABCB10     ------------------------------------------------------------

hABCB7      ---------------------------------MALLAMHSWRWAAAAAAFEKRRHSAIL   27
hABCB6      LAGRVGTARGAPLPSYLLLASVLESLAGACGLWLLVVERSQARQRLAMGIWIKFRHSPGL   150
hABCB10     ------MRGPPAWPLRLLEPPSPAEPGRLLPVACVWAAASRVPGSLSPFTGLRPARLWGA   54
                                                          :     :  :

hABCB7      IRPLVSVSGSGPQWRPHQLGALGTARAYQIPESLKSITWQRLGKGNSGQFLDAAKALQVW   87
hABCB6      LLLWTVAFAAENLALVSWNSPQWWWARADLGQQVQFSLWVLRYVVSGGLFVLGLWAPGLR   210
hABCB10     GPALLWGVGAARRWRSGCRGGGPGASRGVLGLARLLGLWARGPGSCRCGAFAGPGAPRLP
                    .:         .         :        *           . .  *  :
                                                         [==========]
hABCB7      PLIEKRTCWHG--HAGGGLHTDPKEGLKDVDTRKIIKAMLSYVWPKD--RPDLRARVAIS   143
hABCB6      PQSYTLQVHEEDQDVERSQVRSAAQQSTWRDFGRKLRLLSGYLWPRG--SPALQLVVLIC   268
hABCB10     RARFPGGPAAAAWAGDEAWRRGPAAPPGDKGRLRPAAAGLPEARKLLGLAYPERRRLAAA   174
                             .   ..       .  :                   :  :  .
            [=========TM1==========]---------------[==================]
hABCB7      LGFLGGAKAMNIVVPFMFKYAVDSLNQMSGNMLNLSDAPNTVATMATAVLIGYGVSRAGA   203
hABCB6      LGLMGLERALNVLVPIFYRNIVNLLTEKAP---WNSLAWTVTSYVFLKFLQGGGTG--ST   323
hABCB10     VGFLTMSSVISMSAPFFLGKIIDVIYTNPT---------VDYSDNLTRLCLGLSAVFLCG   225
            :*::    .:.: .*::    :: :   .             :     .  * ..
            [=============TM2=============]--[=ICH1=]--[==================]
hABCB7      AFFNEVRNAVFGKVAQNSIRRIAKNVFLHLHNLDLGFHLSRQTGALSKAIDRGTRGISFV   263
hABCB6      GFVSNLRTFLWIRVQQFTSRRVELLIFSHLHELSLRWHLGRRTGEVLRIADRGTSSVTGL   383
hABCB10     AAANAIRVYLMQTSGQRIVNRLRTSLFSSILRQEVAFFDKTRTGELINRLSSDTALLGRS   285
            .  . :*  :     *   .*:   :*  : . .: :.   :** : .  . .*  :
            [=========TM3==========]-[=============TM4================]
hABCB7      LSALVFNLLPIMFEVMLVSGVLYYKCGAQFALVTLGTLGTYTAFTVAVTRWRTRFRIEMN   323
hABCB6      LSYLVFNVIPTLADIIIGIIYFSMFFNAWFGLIVFLCMSLYLTLTIVVTEWRTKFRRAMN   443
hABCB10     VTENLSDGLRAGAQASVGISMMFFVS-PNLATFVLSVVPPVSIIAVIYGRYLRKLTKVTQ   344
            ::  : : :    :  :    :     . :. ..:  :     :::   .:  ::    :
            [===========]-[=ICH2=]-[=============TM5==================]
hABCB7      KADNDAGNAAIDSLLNYETVKYFNNERYEAQRYDGFLKTYETASLKSTSTLAMLNFGQSA   383
hABCB6      TQENATRARAVDSLLNFETVKYYNAESYEVERYREAIIKYQGLEWKSSASLVLLNQTQNL   503
hABCB10     DSLAQATQLAEERIGNVRTVRAFGKEMTEIEKYASKVDHVMQLARKEAFARAGFFGATGL   404
                 :   * : : * .**: :. *  * ::*   :        *.: : . :    .
            [=================]---[===============TM6==================]
hABCB7      IFSVGLTAIMVLASQGIVAGTLTVGDLVMVNGLLFQLSLPLNFLGTVYRETRQALIDMNT   443
hABCB6      VIGLGLLAGSLLCAYFVTEQKLQVGDYVLFGTYIIQLYMPLNWFGTYYRMIQTNFIDMEN   563
hABCB10     SGNLIVLSVLYKGGLLMGSAHMTVGELSSFLMYAFWVGISIGGLSSFYSELMKGLGAGGR   464
              .: : :     .  :    :  **:   .    : : :.:. :.: *      :
```

**Supplementary Figure 1. Sequence alignment of human ABCB6, ABCB7 and ABCB10.** The secondary structure (above) refers to human ABCB7. Conserved NBD motifs are indicated in yellow (Walker A), red (Signature motif), and green (Walker B).

```
                                                   NBD
hABCB7    LFTLLKVDTQIKDKVMASPLQITPQTATVAFDNVHFEYIE--GQKVLSGISFEVPAGKKV   501
hABCB6    MFDLLKEETEVKDLPGAGPLRFQ--KGRIEFENVHFSYAD--GRETLQDVSFTVMPGQTL   619
hABCB10   LWELLEREPKLPFNEGVILNEKS-FQGALEFKNVHFAYPARPEVPIFQDFSLSIPSGSVT   523
          :: **: :.::     .   .     . : *.**** *        :...*: : .*.

              Walker A
hABCB7    AIVGGSGSGKSTIVRLLFRFYEPQKGSIYLAGQNIQDVSLESLRRAVGVVPQDAVLFHNT   561
hABCB6    ALVGPSGAGKSTILRLLFRFYDISSGCIRIDGQDISQVTQASLRSHIGVVPQDTVLFNDT   679
hABCB10   ALVGPSGSGKSTVLSLLLRLYDPASGTISLDGHDIRQLNPVWLRSKIGTVSQEPILFSCS   583
          *:** **:****:: **:*:*:  .* * : *::* ::.   **  :*.*.*:.:**  :

                                                        Signature motif
hABCB7    IYYNLLYGN---ISASPEEVYAVAKLAGLHDAILRMPHGYDTQVGERGLKLSGGEKQRVA   618
hABCB6    IADNIRYGR---VTAGNDEVEAAAQAAGIHDAIMAFPEGYRTQVGERGLKLSGGEKQRVA   736
hABCB10   IAENIAYGADDPSSVTAEEIQRVAEVANAVAFIRNFPQGFNTVVGEKGVLLSGGQKQRIA   643
          *  *: **     :.  :*:  .*: *.    *  :*.*: * ***:*: ****:***:*

                    Walker B
hABCB7    IARAILKDPPVILYDEATSSLDSITEETILGAMKDVVKHRTSIFIAHRLSTVVDADEIIV   678
hABCB6    IARTILKAPGIILLDEATSALDTSNERAIQASLAKVCANRTTIVVAHRLSTVVNADQILV   796
hABCB10   IARALLKNPKILLLDEATSALDAENEYLVQEALDRLMDGRTVLVIAHRLSTIKNANMVAV   703
          ***::** * ::* *****:**: .*  :  ::  :   ** :.:******: :*: : *

hABCB7    LDQGKVAERGTHHGLLANPHSIYSEMWHTQSSRVQNHDNPKWEAKKENISKEEERKKLQE   738
hABCB6    IKDGCIVERGRHEALLS-RGGVYADMWQLQQGQEETSEDTKPQTMER-------------   842
hABCB10   LDQGKITEYGKHEELLSKPNGIYRKLMNKQSFISA-------------------------   738
          :.:* :.* * *. **:   .:* .: : *.

hABCB7    EIVNSVKGCGNCSC    752
hABCB6    --------------
hABCB10   --------------
```

**Supplementary Figure 1 (continued).**

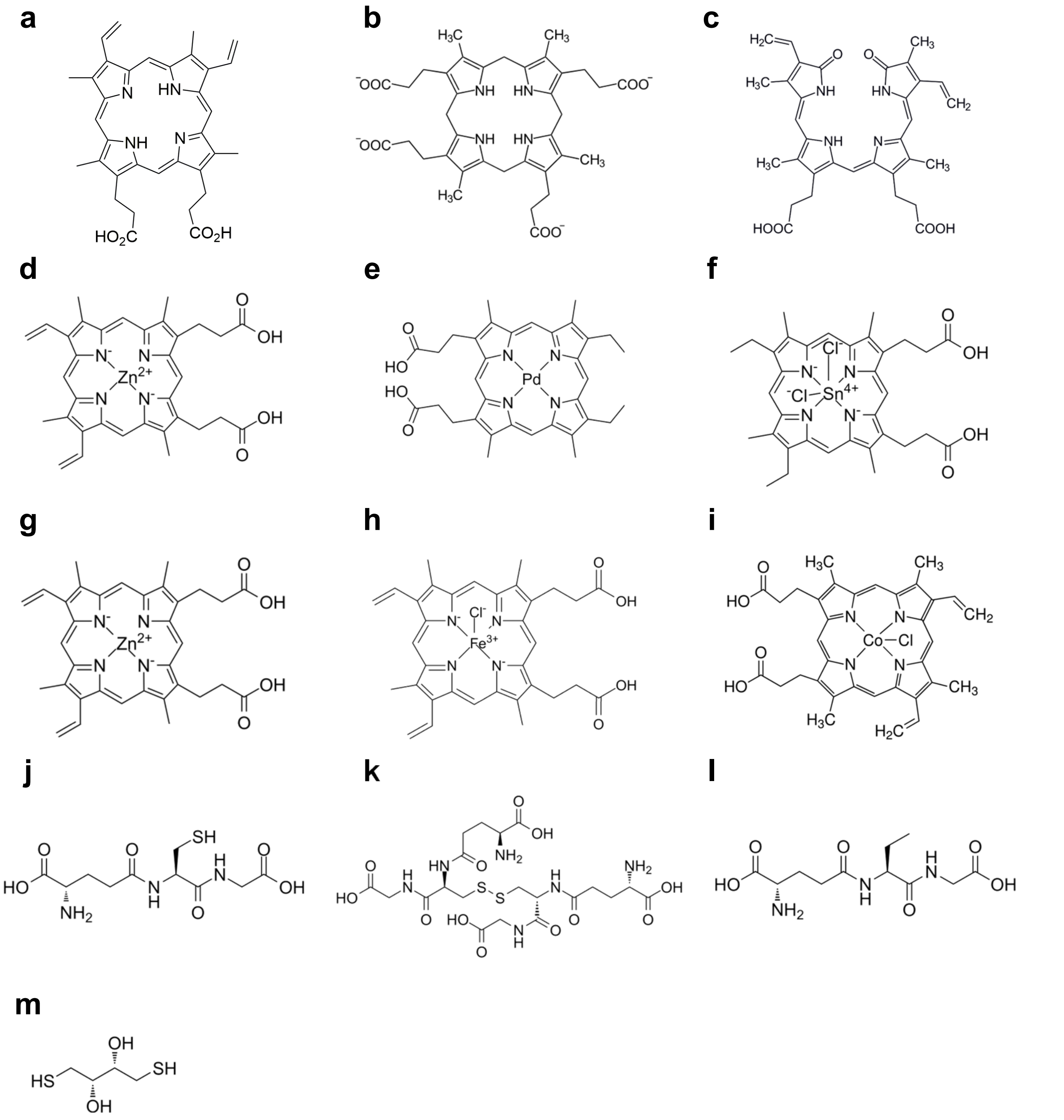

**Supplementary Figure 2. Chemical structures of the potential ABCB7 substrates tested in this study.** (a) PPIX (Protoporphyrin IX). (b) CPⅢ (Coproporphyrinogen Ⅲ). (c) BV (Biliverdin). (d) ZnMP (Zinc(Ⅱ) Mesoporphyrin IX). (e) PdMP (Pd(Ⅱ) Mesoporphyrin IX). (f) SnMP (Tin Mesoporphyrin IX). (g) ZnPP (Zinc protoporphyrin). (h) Hemin (Iron (III) Protoporphyrin IX chloride). (i) CoPP (Cobalt (III) Protoporphyrin IX chloride). (j) GSH (Reduced Glutathione). (k) GSSG (Oxidised Glutathione). (l) OPT (Ophthalmic acid). (m) DTT (Dithiothreitol).

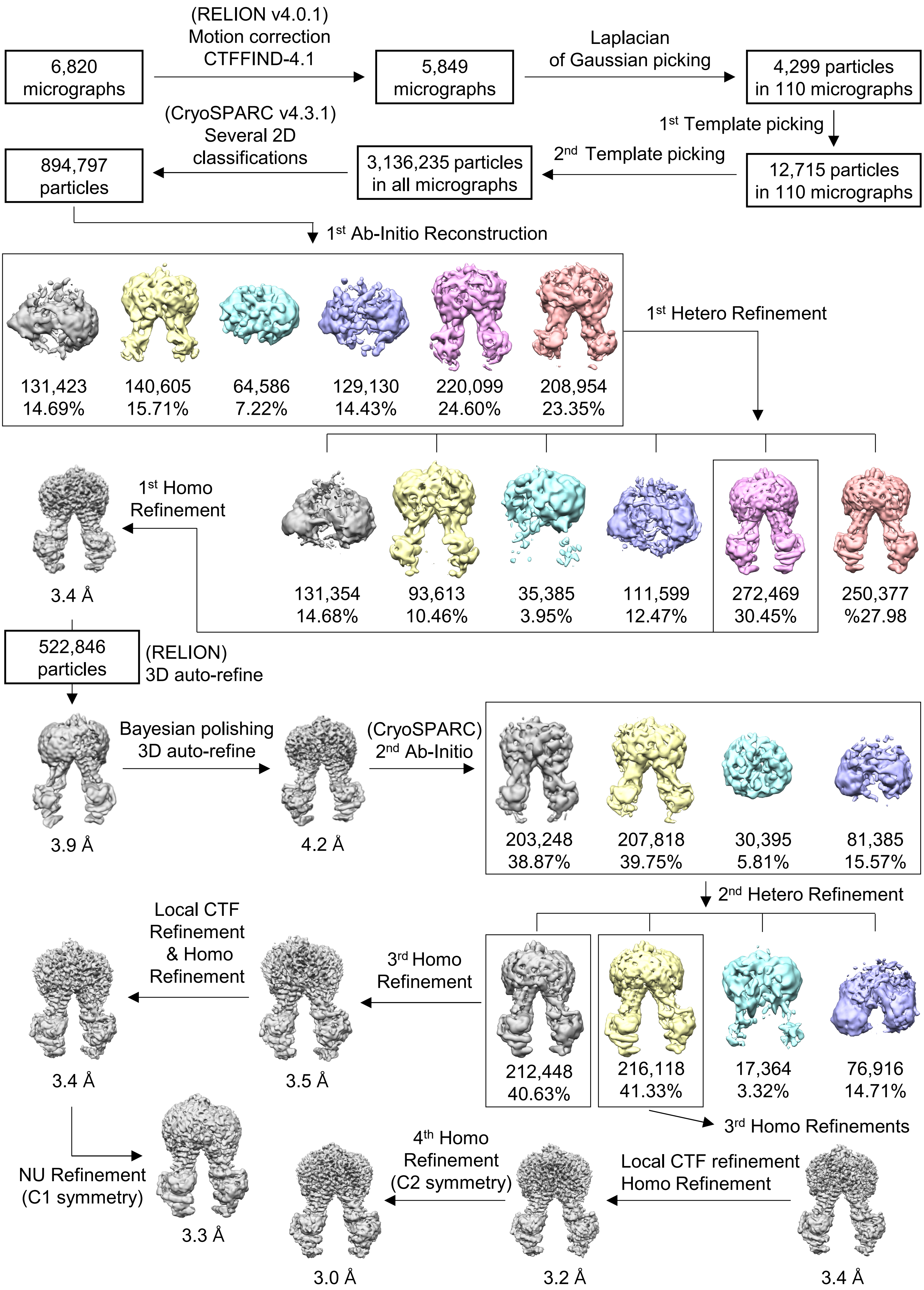


**Supplementary Figure 3. Cryo-EM data-processing workflow for apo ABCB7.** The main steps of cryo-EM data processing are shown.

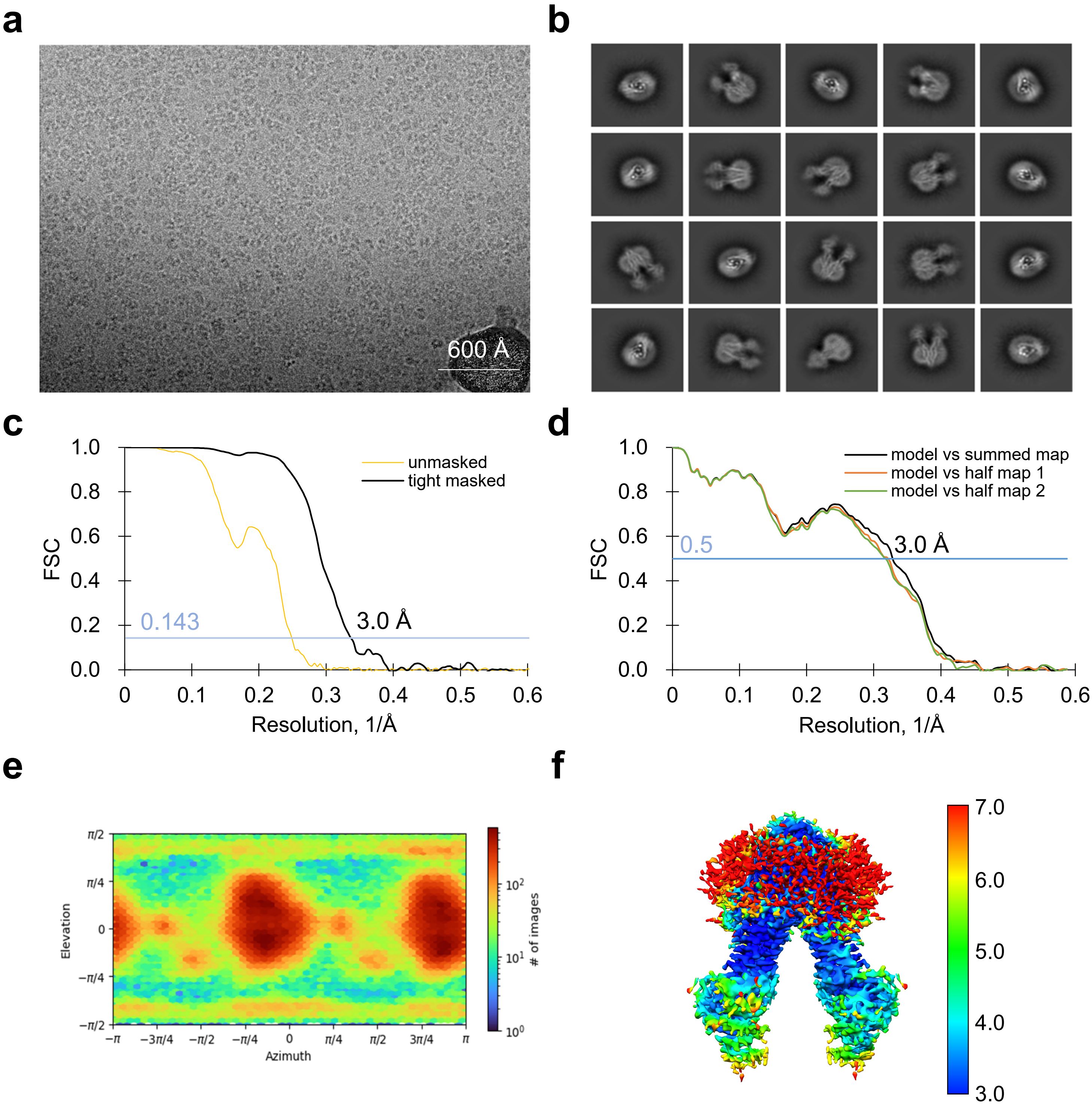


**Supplementary Figure 4. Cryo-EM data and model quality analysis of apo ABCB7.** (a) Representative micrograph. (b) Selected 2D class averages. (c) Gold-standard Fourier Shell correlation (FSC) curve. The resolution (blue line) was estimated based on an FSC cut-off of 0.143. (d) FSC curves calculated between the refined structure and the half map used for refinement (orange), the other half map (green) and the summed map (black). (e) Particle distribution plot for the final 3D reconstruction. (f) Density map colored by local resolution estimation from 3 Å (blue) to 7 Å (red).

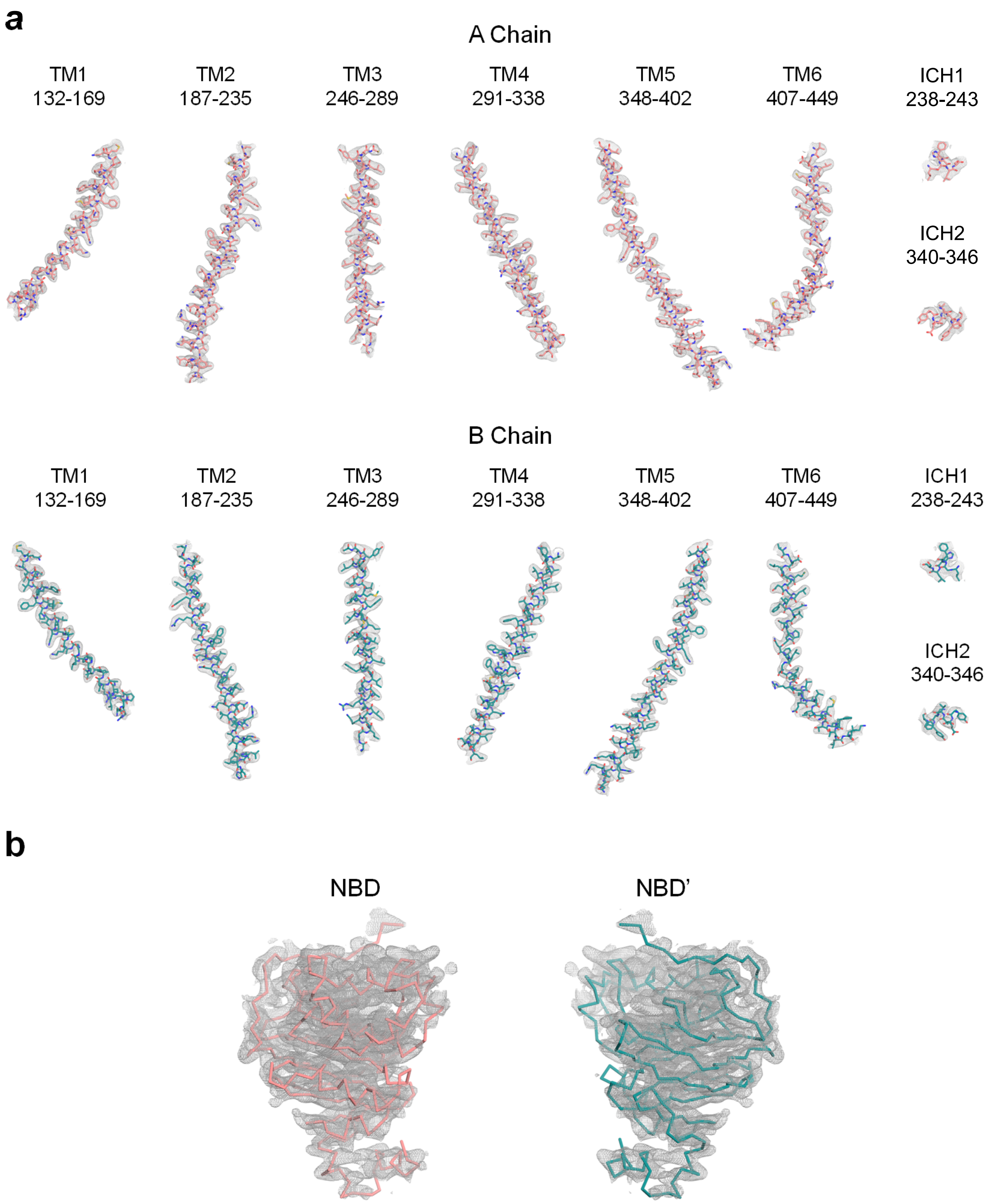


**Supplementary Figure 5. Cryo-EM density maps of apo ABCB7.** (a) The amino acid residues of each TM helix are shown. The EM density (grey mesh) is contoured at the 4 $\sigma$ level. (b) The cryo-EM density of the NBD region.

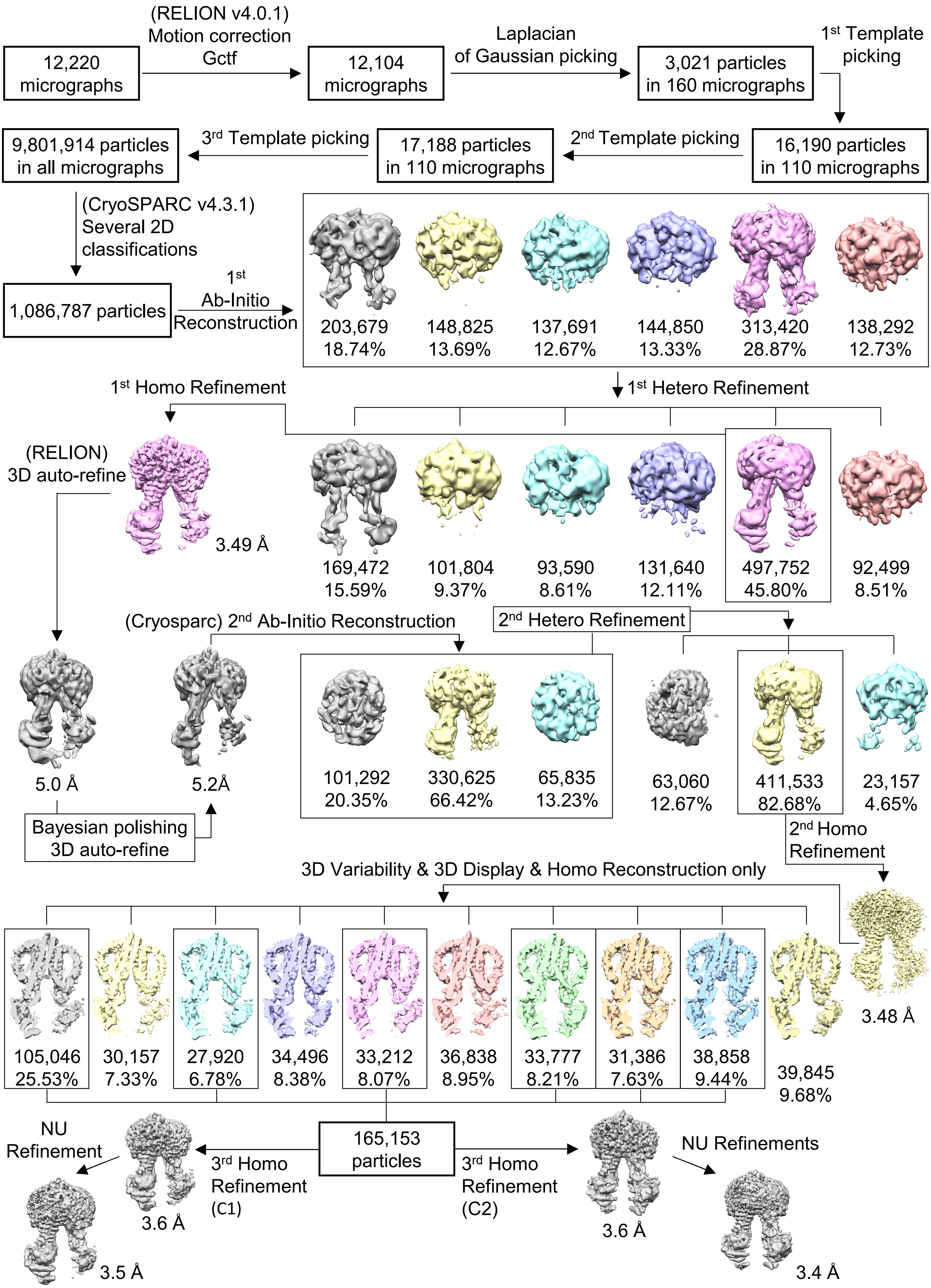


**Supplementary Figure 6. The cryo-EM data-processing workflow for CoPP:GSH-bound ABCB7.** The main steps of the cryo-EM data processing pipeline for the substrate-bound sample are shown.

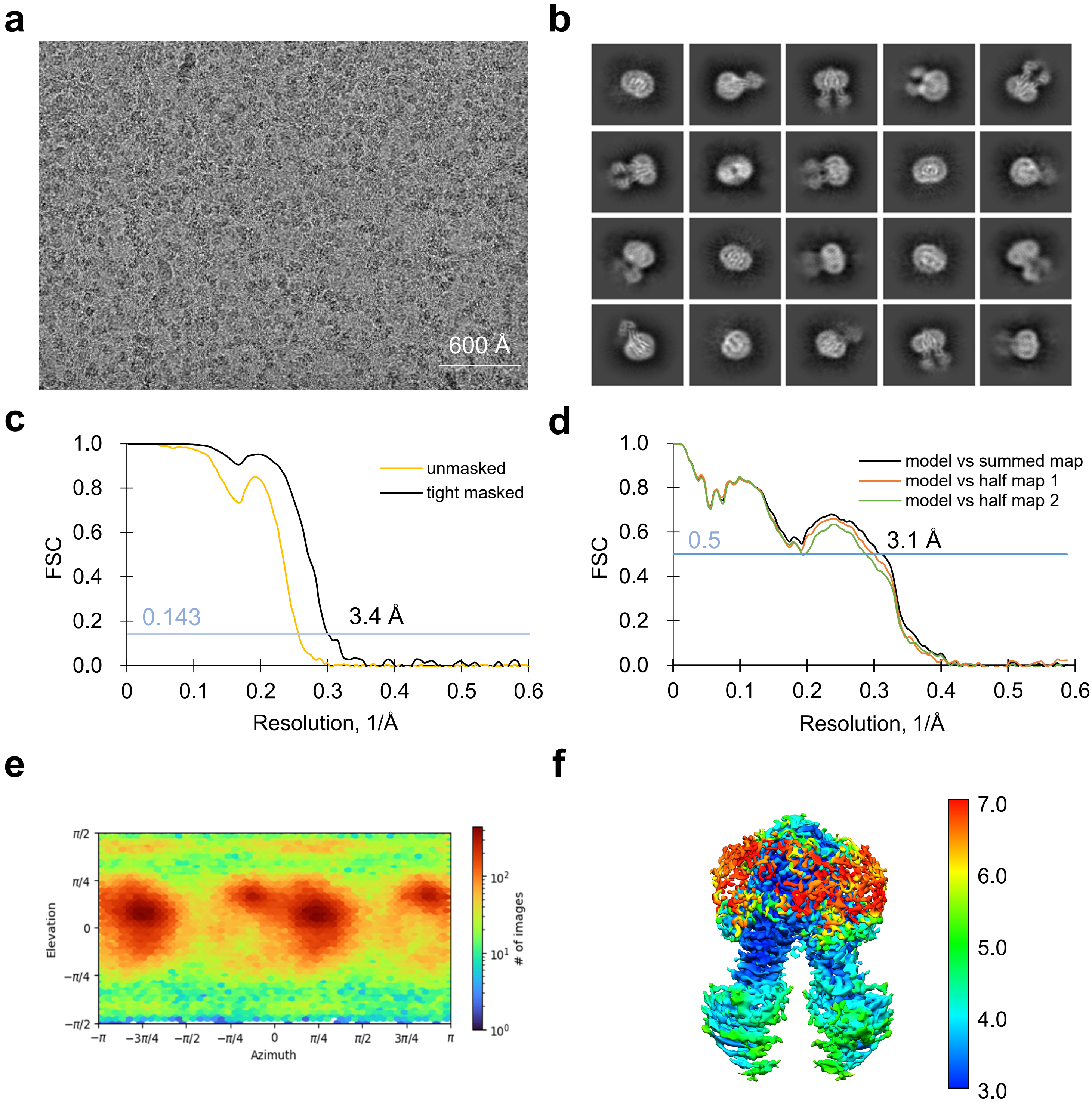


**Supplementary Figure 7. Cryo-EM data and model quality analysis of CoPP:GSH-bound ABCB7.** (a) Representative micrograph. (b) Selected 2D class averages. (c) Gold-standard Fourier Shell correlation (FSC) curve. The resolution estimation (blue line) was calculated based on an FSC cut-off of 0.143. (d) FSC curves calculated between the refined structure and the half map used for refinement (orange), the other half map (green) and the summed map (black). (e) Particle distribution plot for the final 3D reconstruction. (f) Density map colored by local resolution estimation from 3 Å (blue) to 7 Å (red).

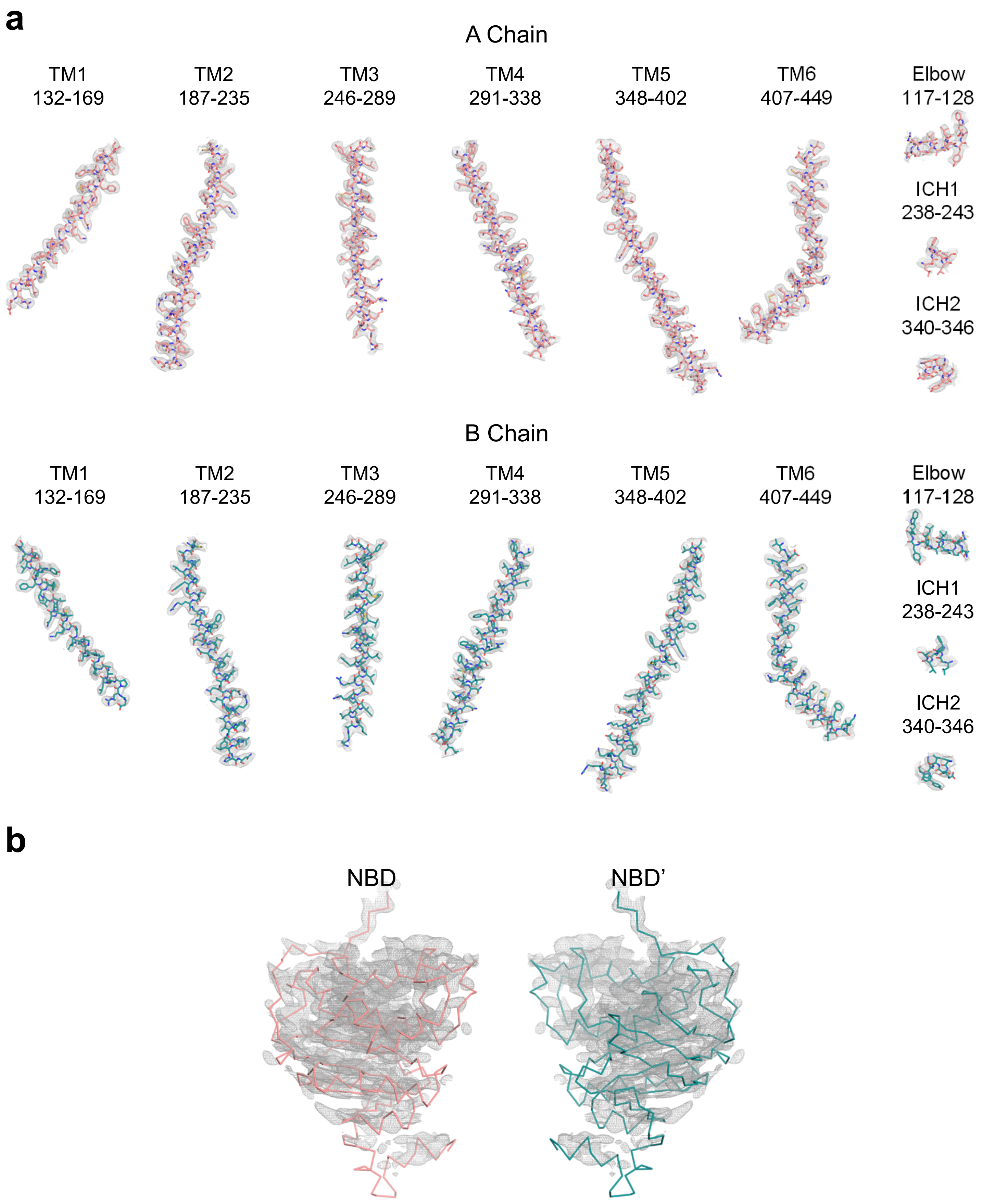


**Supplementary Figure 8. Cryo-EM density maps of CoPP:GSH-bound ABCB7.** (a) The amino acid residues of each TM helix are shown. The EM density (grey mesh) is contoured at 4 $\sigma$ level. (b) The cryo-EM density of the NBD region.

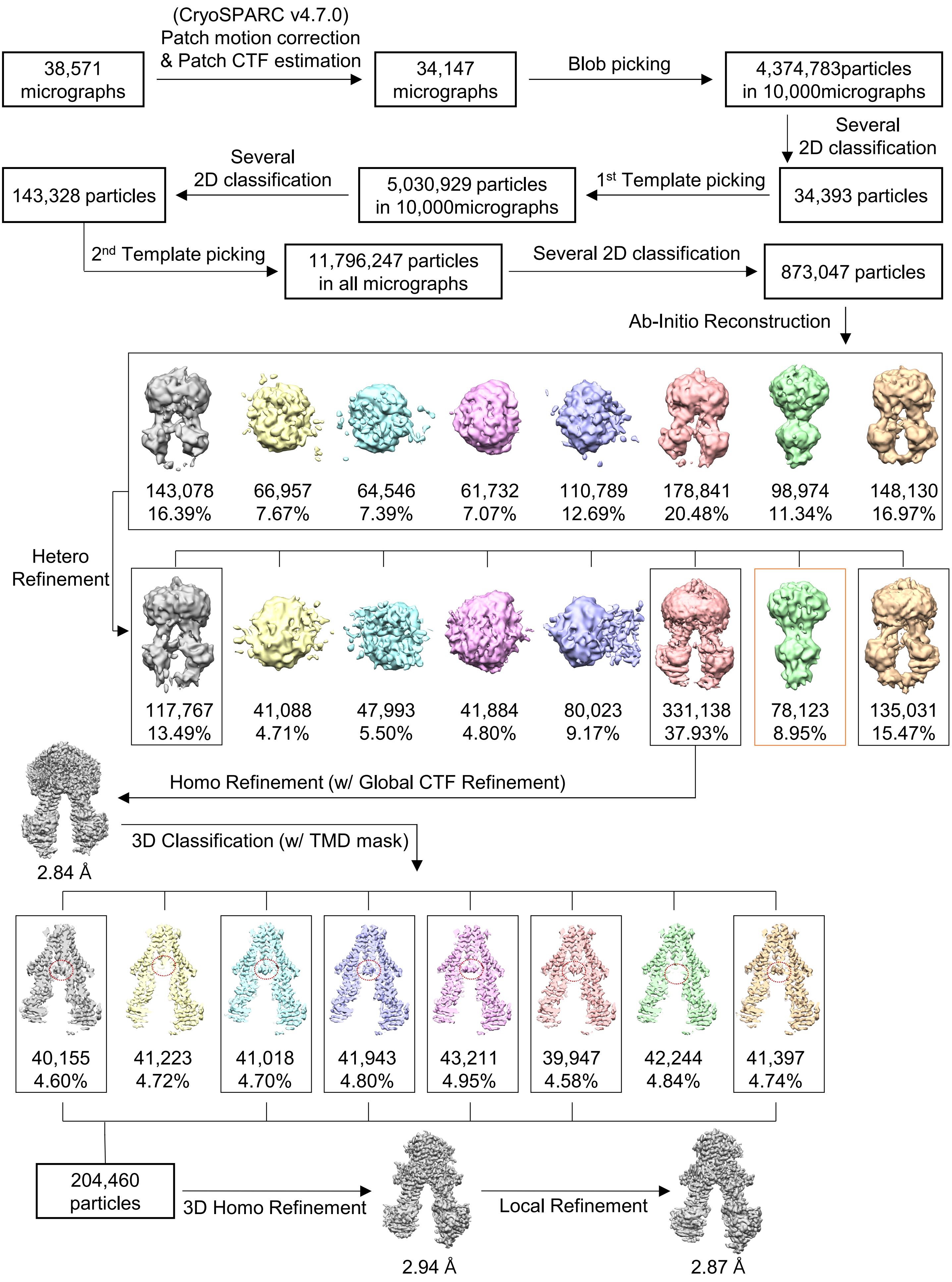


**Supplementary Figure 9. Cryo-EM data-processing workflow for CoPP:GSH/ADP·$VO_4$-bound ABCB7.** The main steps of the cryo-EM data processing pipeline for the CoPP:GSH/ADP·$VO_4$-bound sample are shown. The 3D class corresponding to the inward-facing and occluded state is highlighted by black and red boxes, respectively.

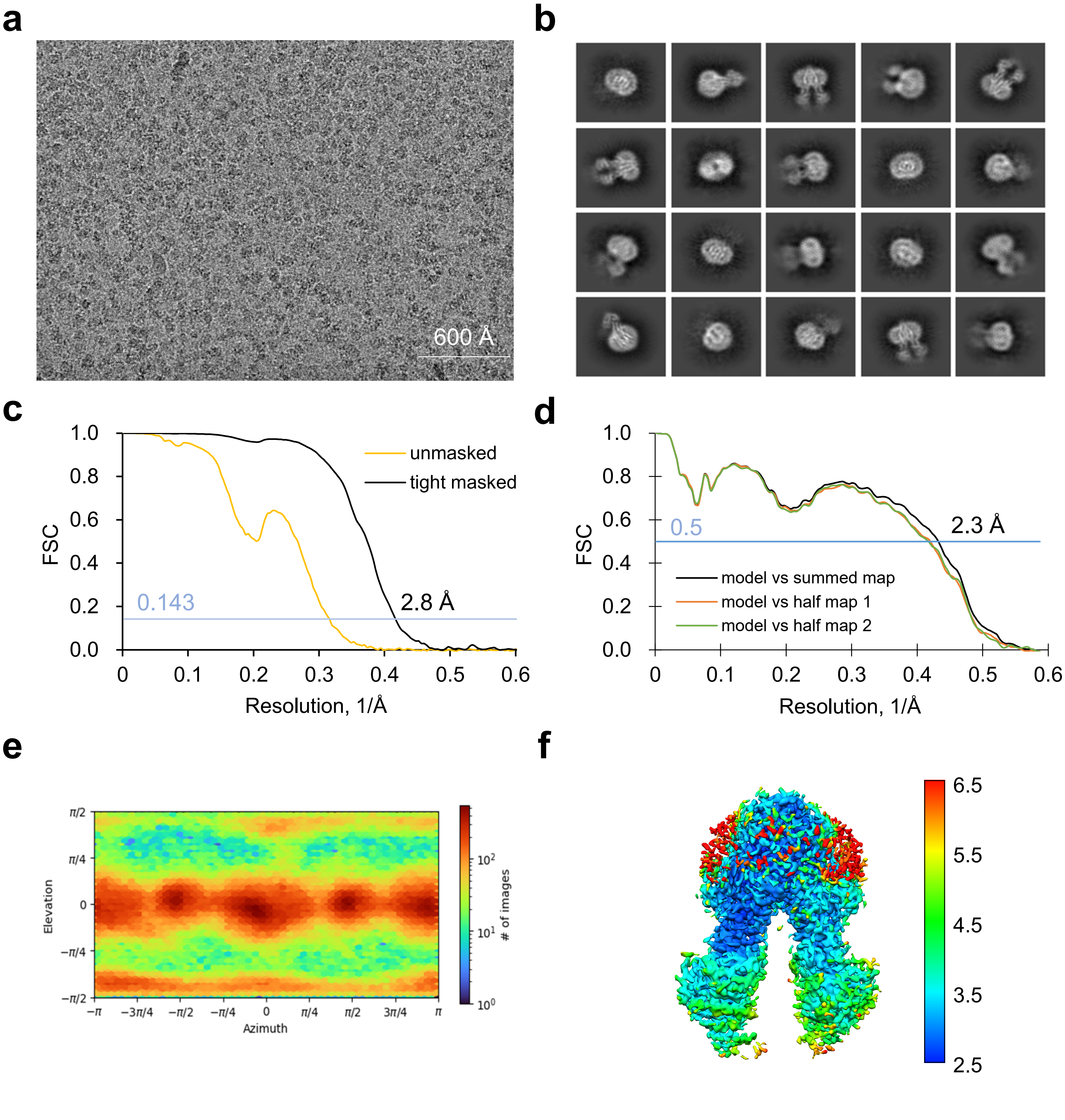


**Supplementary Figure 10. Cryo-EM data and model quality analysis of CoPP:GSH/ADP·$VO_4$-bound ABCB7.** (a) Representative micrograph. (b) Selected 2D class averages. (c) Gold-standard Fourier Shell correlation (FSC) curve. The resolution (blue line) was estimated based on an FSC cut-off of 0.143. (d) FSC curves calculated between the refined structure and the half map used for refinement (orange), the other half map (green) and the summed map (black). (e) Particle distribution plot for the final 3D reconstruction. (f) Density map colored by local resolution estimation from 2.5 Å (blue) to 6.5 Å (red).

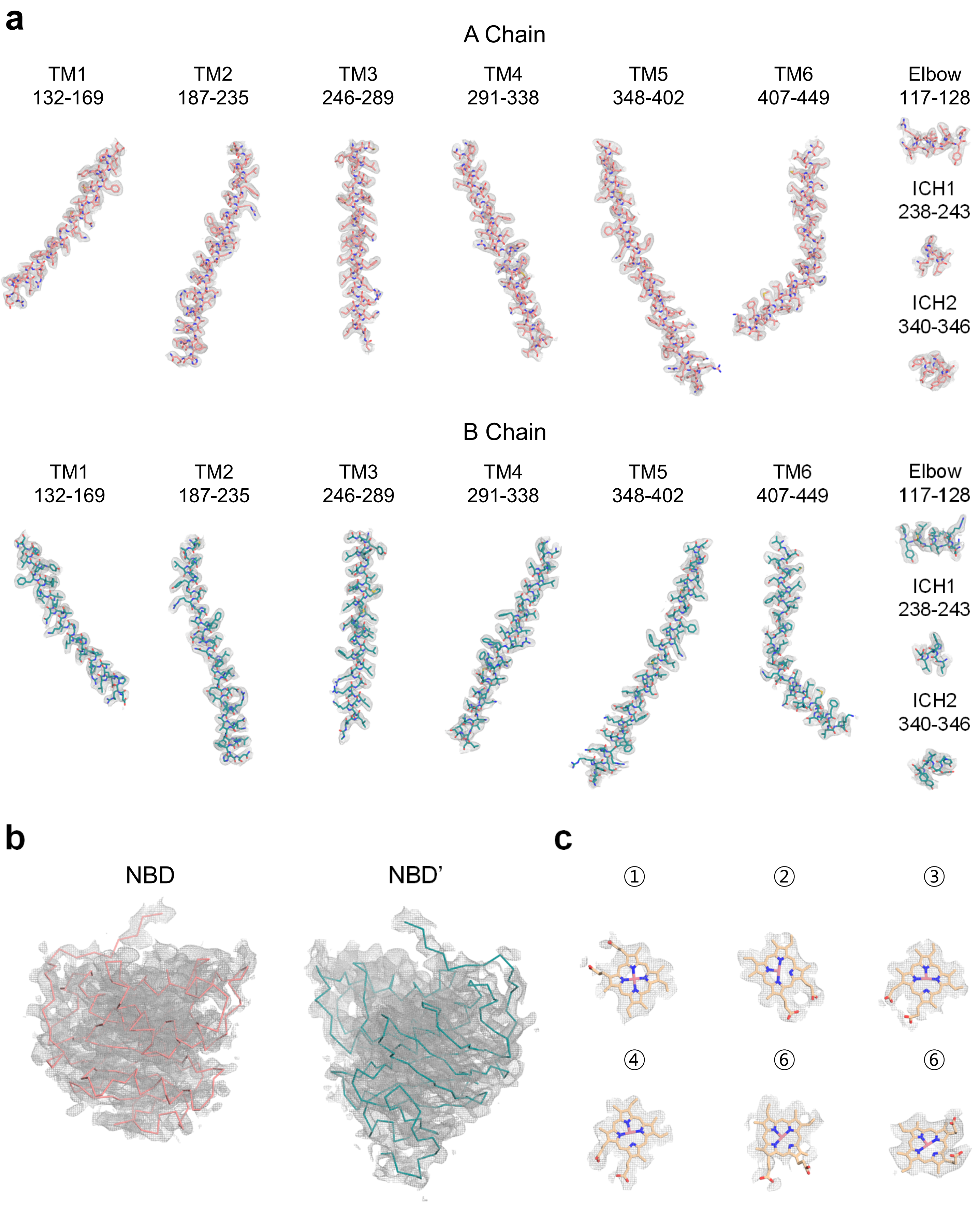


**Supplementary Figure 11. Cryo-EM density maps of CoPP:GSH/ADP·$VO_4$-bound ABCB7.** (a) The amino acid residues of each TM helix are shown. The EM density (grey mesh) is contoured at 4 σ level. (b, c) Cryo-EM densities of the NBD region (b), and the CoPP molecules (c) corresponding to those shown in Fig. 4. The densities are contoured at 4 σ and 3 σ levels, respectively.

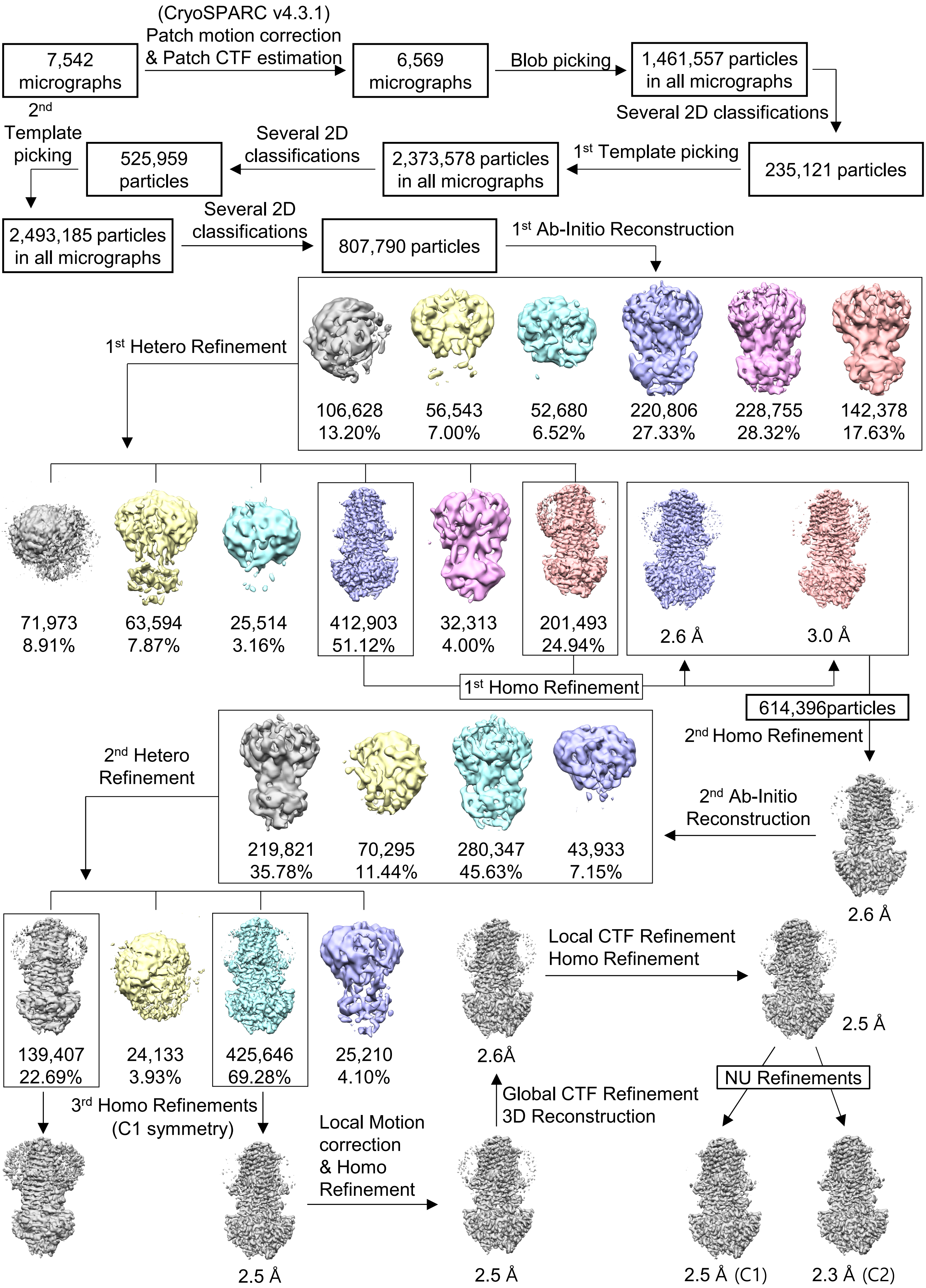


**Supplementary Figure 12. Cryo-EM data-processing workflow for ATP-bound ABCB7 (E634Q) in the occluded state.** The main steps of the cryo-EM data processing pipeline for the ATP-bound sample are shown.

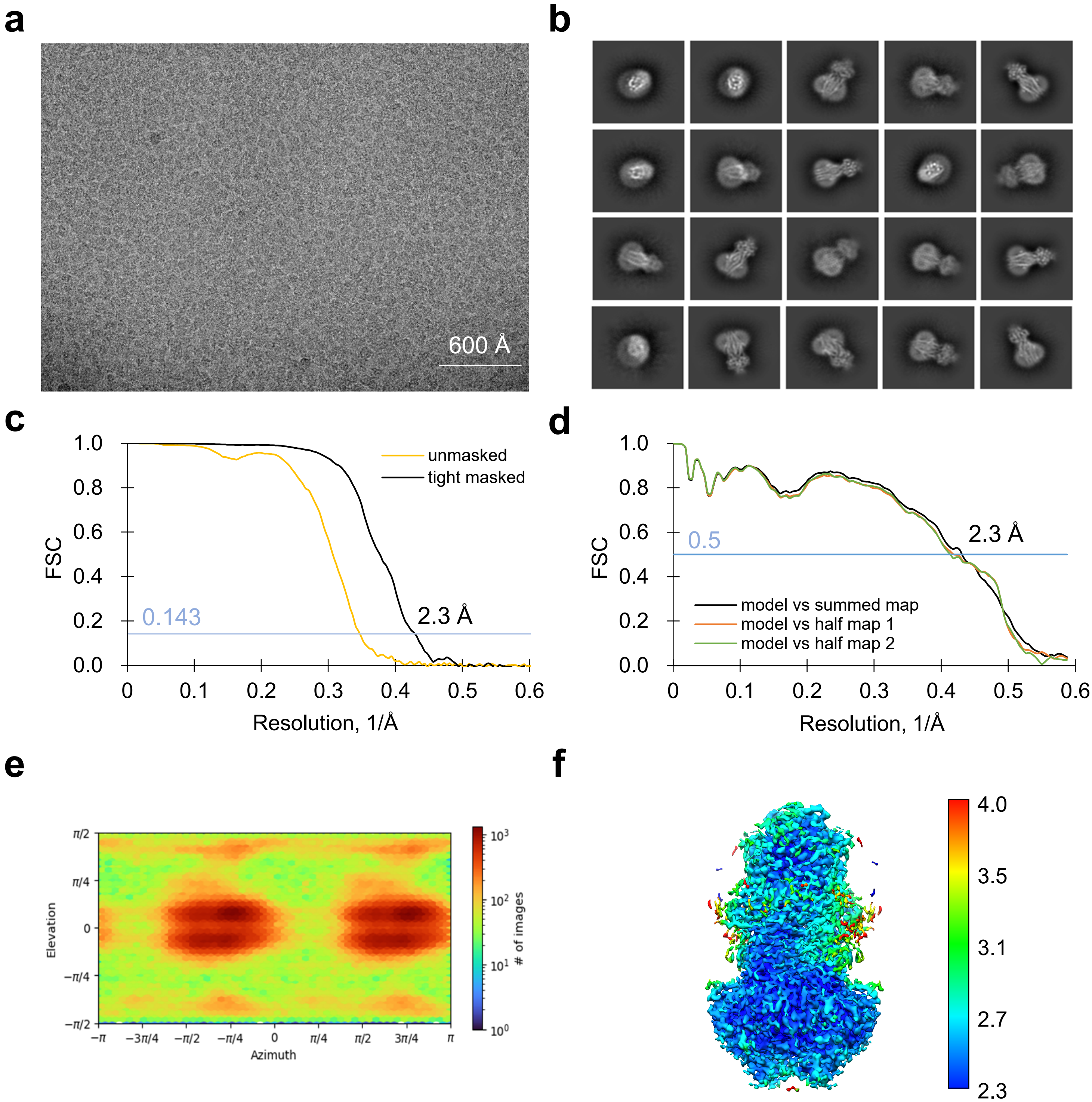


**Supplementary Figure 13. Cryo-EM data and model quality analysis of ATP-bound ABCB7 (E634Q) in the occluded state.** (a) Representative micrograph. (b) Selected 2D class averages. (c) Gold-standard Fourier Shell correlation (FSC) curve. The resolution estimation (blue line) was calculated based on an FSC cut-off of 0.143. (d) FSC curves calculated between the refined structure and the half map used for refinement (orange), the other half map (green) and the summed map (black). (e) Particle distribution plot for the final 3D reconstruction. (f) Density map colored by local resolution estimation from 2.3 Å (blue) to 4.0 Å (red).

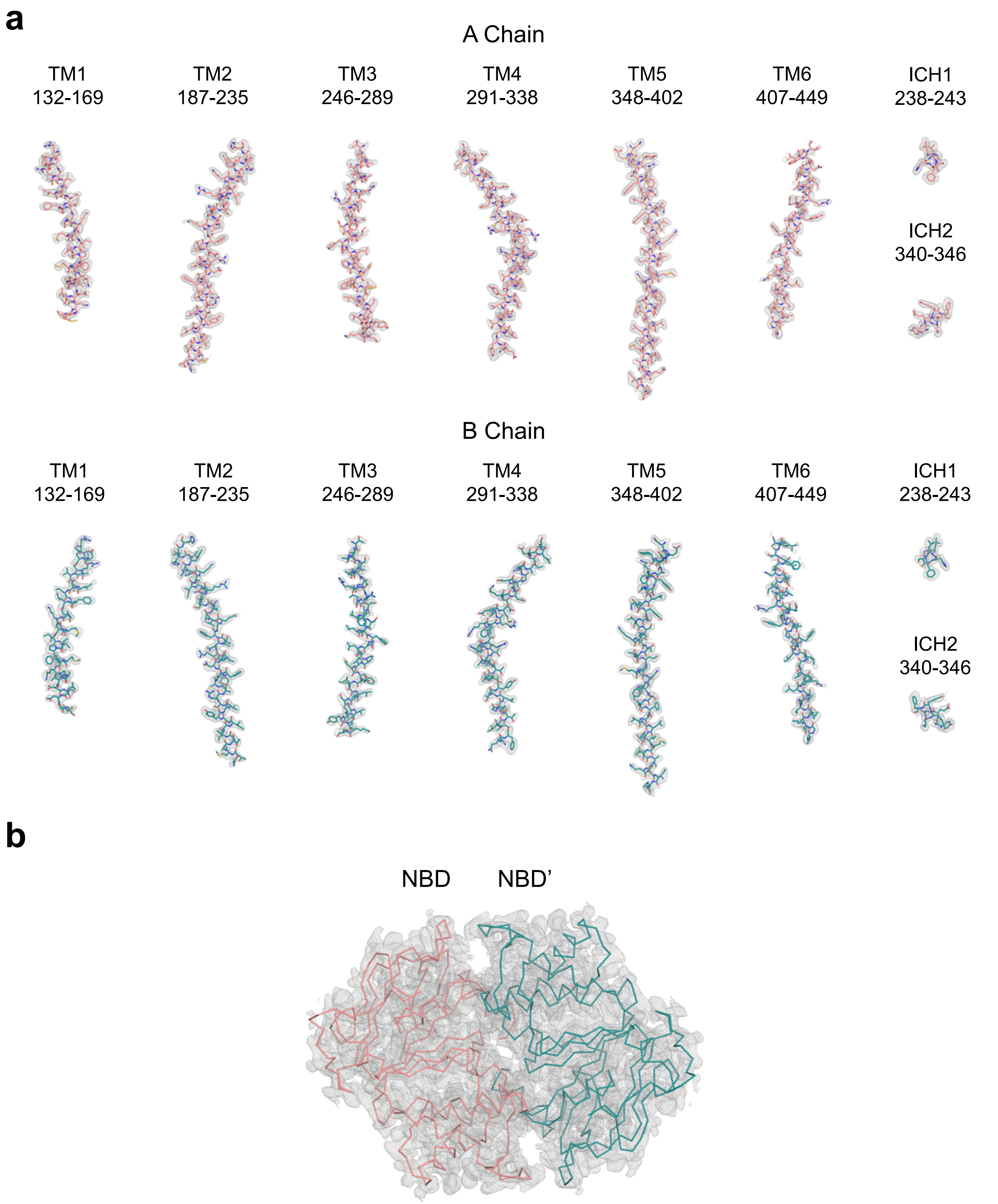


**Supplementary Figure 14. Cryo-EM density maps of ATP-bound ABCB7 (E634Q) in the occluded state.** (a) The amino acid residues of each TM helix are shown. The EM density (grey mesh) is contoured at 4 σ level. (b) Cryo-EM density of the NBD region.

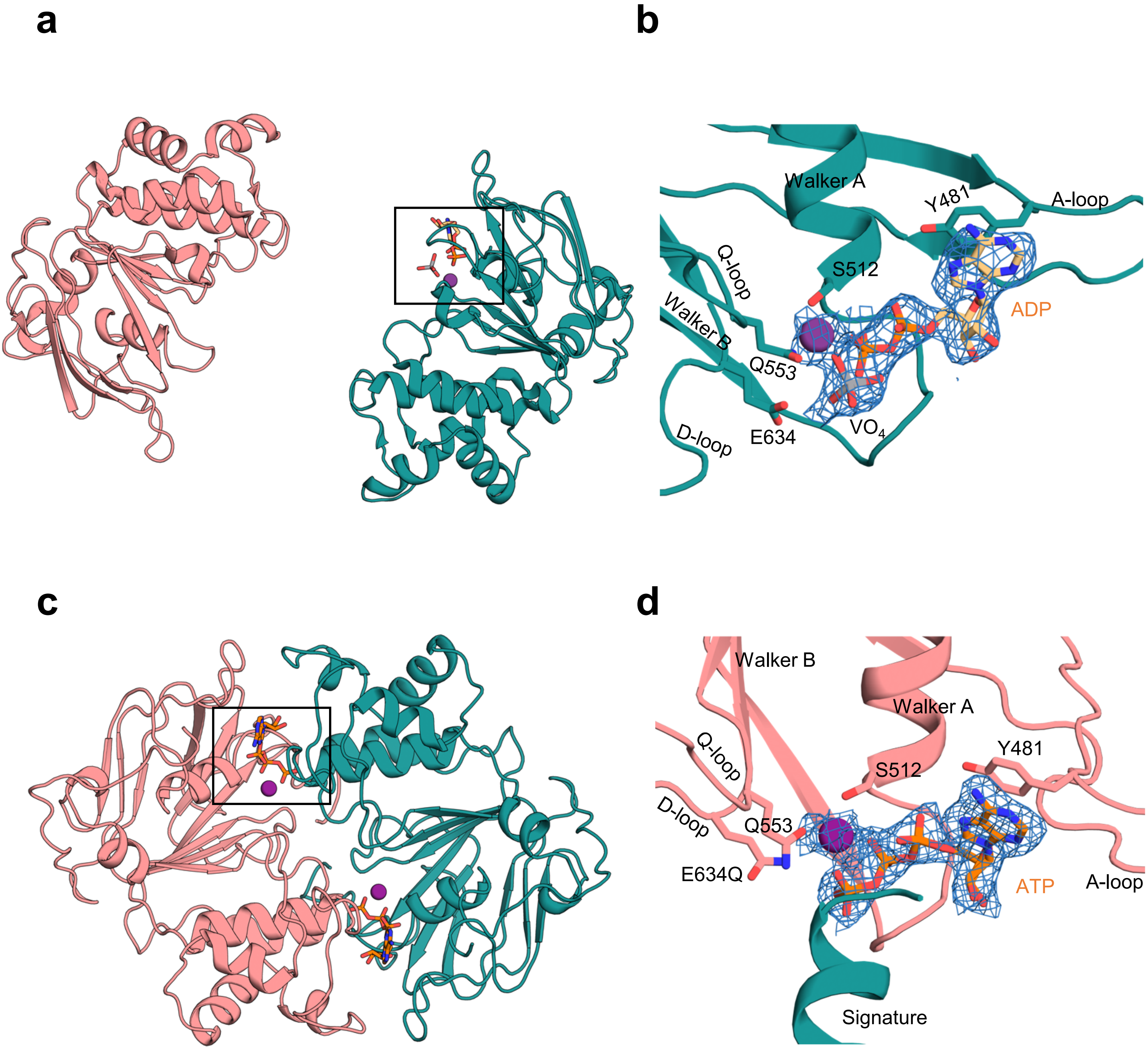


**Supplementary Figure 15. The NBD structures of ADP·VO4-bound and ATP-bound human ABCB7.** (a) Overall NBD structure of ADP·$VO_4$-bound ABCB7 (wild-type) in a post-hydrolysis conformation. The two NBDs are colored pink and teal. The bound ADP·$VO_4$ and $Mg^{2+}$ are shown as sticks and spheres, respectively. (b) Close-up view of the ADP·$VO_4$ binding site. The EM density (blue mesh) of the ADP·$VO_4$ and $Mg^{2+}$ are shown at the 5 $\sigma$ level. (c) Overall NBD structure of ATP-bound ABCB7 (E634Q) in the occluded state. The bound ATP and $Mg^{2+}$ are shown as sticks and spheres, respectively. (d) Close-up view of the ATP binding site in the occluded state. The EM density (blue mesh) of $Mg^{2+}$/ATP is shown at the 5 $\sigma$ level.

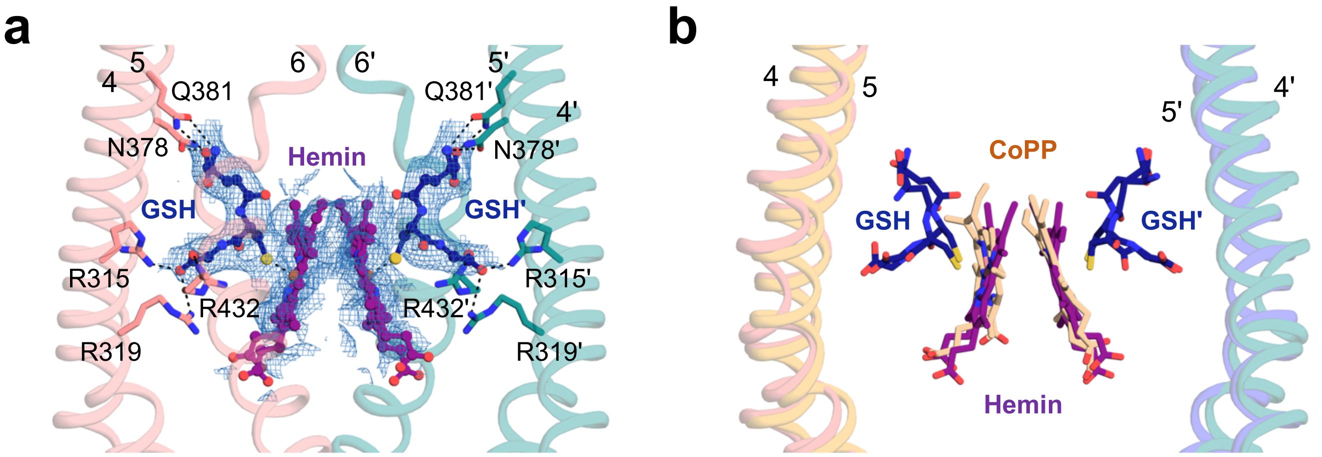


**Supplementary Figure 16. Structural comparison of the CoPP:GSH and hemin:GSH complexes bound to ABCB7.** (a) Close-up view of the hemin:GSH-binding site. Cryo-EM density maps of bound hemin and GSH are shown as meshes contoured at 4σ. (b) Structural comparison of the CoPP:GSH-bound and hemin:GSH-bound ABCB7 complexes.

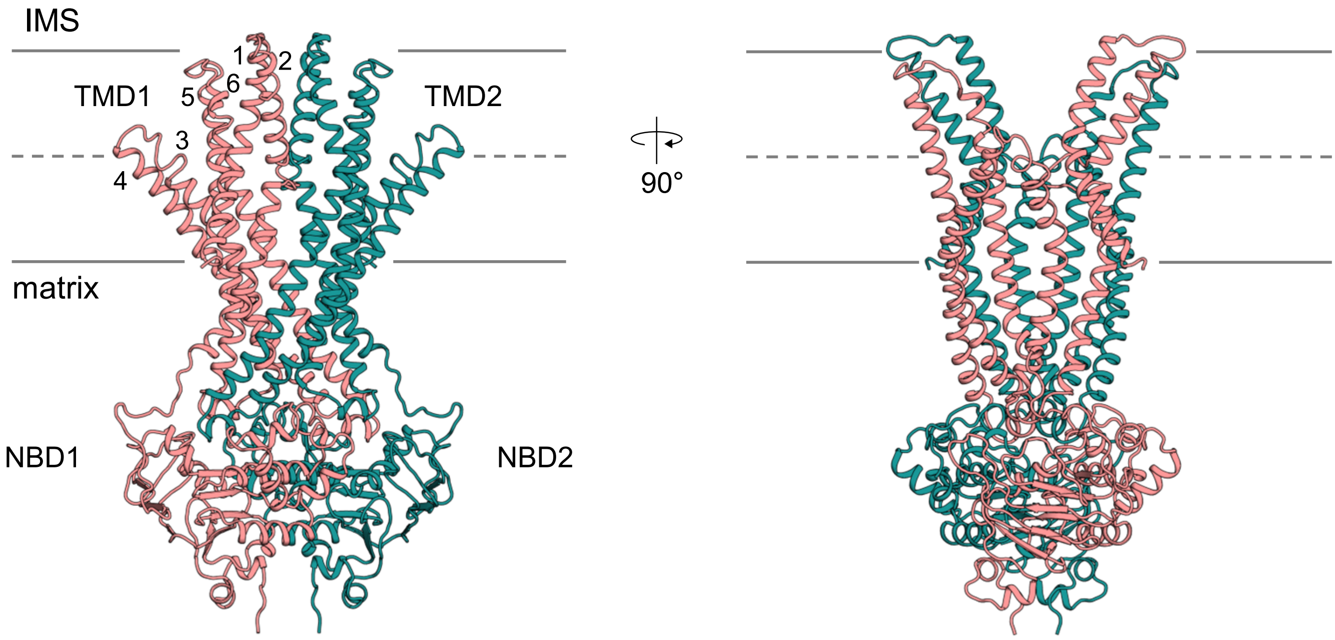


**Supplementary Figure 17. Homology model of outward-facing ABCB7 generated by MODELLER.** The overall structure of outward-facing ABCB7 showing the predicted TMD arrangement and NBD dimerization.

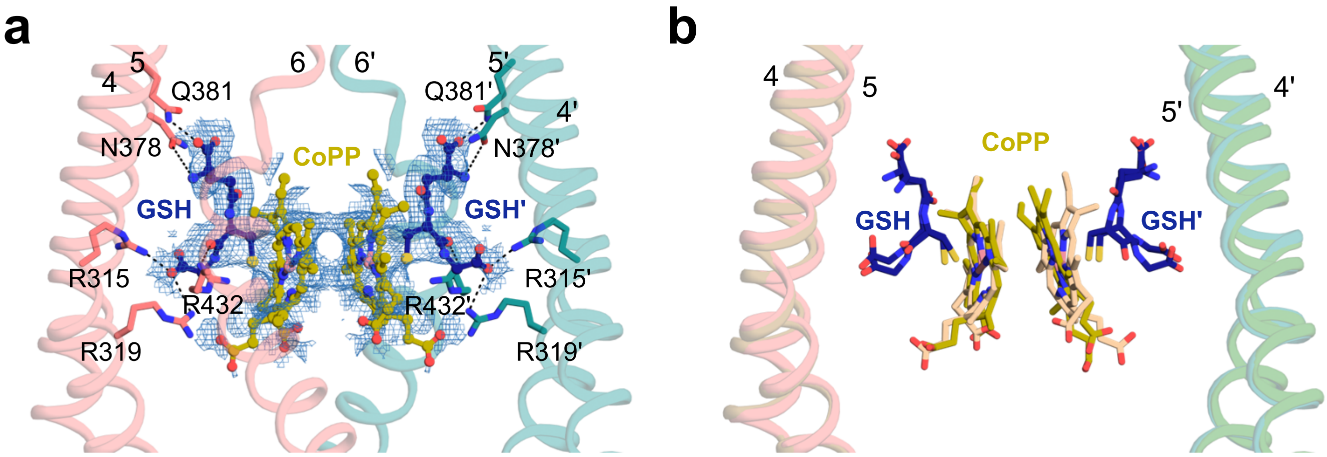


**Supplementary Figure 18. Structural comparison of ABCB7 bound to CoPP at low concentrations.** (a) Close-up view of the substrate-binding cavity in the presence of 0.1 mM CoPP and 1 mM GSH. The EM density maps for bound CoPP and GSH are contoured at 6 σ and 4 σ, respectively. (b) Structural comparison of ABCB7 complexes prepared with 1 mM and 0.1 mM CoPP (both in the presence of 1 mM GSH).

**Supplementary Table 1. Cryo-EM data collection, refinement and validation statistics**

| | **Apo**<br>EMDB-69144<br>PDB ID 23PH | **CoPP:GSH /ADP·$VO_4$**<br>EMDB-69143<br>PDB ID 23PG | **ATP-bound**<br>EMDB-69145<br>PDB ID 23PI |
|---|---|---|---|
| **Data collection and processing** | | | |
| Microscope | Titan Krios G4 with XFEG | Titan Krios G4 with CFEG | Titan Krios G4 with XFEG |
| Camera | K3 | Falcon 4i | K3 |
| Energy filter | BioQuantum | Selectris X | BioQuantum |
| Magnification | 130,000 | 165,000 | 130,000 |
| Voltage (kV) | 300 | 300 | 300 |
| Total movies (no.) | 6,820 | 38,571 | 7,542 |
| Electron exposure (e–/Å$^2$) | 50 | 50 | 50 |
| Defocus range (μm) | -0.8 ~ -2.2 | -0.8 ~ -2.0 | -0.8 ~ -2.0 |
| Pixel size (Å) | 0.673 | 0.760 | 0.673 |
| Symmetry imposed | C2 | C1 | C2 |
| Initial particle images (no.) | 3,136,235 | 11,796,247 | 2,493,185 |
| Final particle images (no.) | 216,118 | 204,460 | 425,646 |
| Map resolution (Å) | 3.0 | 2.8 | 2.3 |
| FSC threshold | 0.143 | 0.143 | 0.143 |
| Map resolution range (Å) | 3.0 ~ 7.0 | 2.8 ~ 6.5 | 2.3 ~ 4.0 |
| | | | |
| **Refinement** | | | |
| Initial model used (PDB code) | 7VGF | 7VGF | ABCB10 |
| Model resolution (Å) | 3.0. | 2.8 | 2.3 |
| Map sharpening *B* factor (Å$^2$) | 116.5 | 83.9 | 83.3 |
| Model composition | | | |
| Non-hydrogen atoms | | | |
| Protein residues | 8,580 | 8,544 | 8,616 |
| Ligands | 70 | 679 | 326 |
| *B* factors (Å$^2$) | | | |
| Protein | 137.2 | 69.6 | 43.6 |
| Ligand | 108.5 | 73.4 | 56.8 |
| R.m.s. deviations | | | |
| Bond lengths (Å) | 0.004 | 0.005 | 0.003 |
| Bond angles (°) | 0.896 | 0.712 | 0.596 |
| Validation | | | |
| MolProbity score | 1.3 | 1.8 | 1.5 |
| Clashscore | 2.5 | 5.7 | 3.8 |
| Poor rotamers (%) | 0.3 | 2.0 | 2.5 |
| Ramachandran plot | | | |
| Favored (%) | 96.3 | 96.6 | 98.2 |
| Allowed (%) | 3.7 | 3.4 | 1.8 |
| Disallowed (%) | 0.0 | 0.0 | 0.0 |
