# Supplementary material for: Cryo-EM structures of human ABCB7 reveal the molecular basis of mitochondrial matrix heme export
Source: Commun Biol. 2026 May 9;9:970. doi: 10.1038/s42003-026-10223-x (PMC13376393; doi:10.1038/s42003-026-10223-x)
Supplement: Supplementary file 2 — Description of Additional Supplementary Materials [file 42003_2026_10223_MOESM2_ESM.pdf]

Description of Additional Supplementary Files

File name: Supplementary Data 1

Description: The Data that support the findings of this study are available within the article.
