# Supplementary material for: Cryo-EM structures of human ABCB7 reveal the molecular basis of mitochondrial matrix heme export
Source: Commun Biol. 2026 May 9;9:970. doi: 10.1038/s42003-026-10223-x (PMC13376393; doi:10.1038/s42003-026-10223-x)
Supplement: Supplementary file 5 — Transparent Peer Review file [file 42003_2026_10223_MOESM5_ESM.pdf]

nature portfolio

Peer Review File

Cryo-EM structures of human ABCB7 reveal the molecular basis of mitochondrial matrix heme export

Corresponding Author: Professor Mi Sun Jin

This file contains all reviewer reports in order by version, followed by all author rebuttals in order by version.

Version 0:

Reviewer comments:

Reviewer #1

(Remarks to the Author)
The authors show that the ATPase activity of ABCB7 is stimulated by Fe- and Co-protoporphyrin IX (PPIX) in the presence of GSH, leading to the proposal that ABCB7 functions as a metalloporphyrin exporter. Using cryo-EM, the authors determined structures of human ABCB7 in multiple conformational states at 2.3 Å resolution. These structures reveal a putative substrate-binding cavity accommodating two stacked CoPP molecules bridged by the thiol groups of two GSH molecules. A conserved phenylalanine residue is proposed to function as a molecular gate for substrate release. The authors further propose that stacked CoPP molecules (up to four) can partition into the lipid bilayer, transfer laterally into the ABCB7 translocation pathway, and be exported.
The study also offers insights into the pathogenic mechanism of the E433K XLSA/A variant, proposing that substitution of a negatively charged Glu with a positively charged Lys causes steric and/or electrostatic interference with substrate binding or release, given the polar nature of porphyrin substrates.
Overall, the paper is of interest and worth sharing with the scientific community. However, several important issues should be addressed to strengthen the conclusions.

Major Comments
1. Physiological relevance of substrate concentrations Based on its similarity to ABCB6 and reported interaction with the heme biosynthetic enzyme FECH, the authors propose that ABCB7 functions as a porphyrin transporter. ABCB7 expressed in insect cells and reconstituted into nanodiscs exhibits a 1.5- and 3.7-fold stimulation of ATPase activity in the presence of hemin and CoPP, respectively, but only in the presence of GSH. A major concern is that the reported Km for CoPP (~16 microM) appears substantially higher than the expected concentration of heme or porphyrin intermediates in the mitochondrial matrix, which is generally thought to be in the low nM range. This discrepancy should at least be discussed. One possibility is that ABCB7 accesses transiently elevated local substrate concentrations near the site of synthesis through direct or indirect interaction with FECH, but this remains speculative and should be clearly framed as such.
2. High porphyrin concentrations used for cryo-EM grid preparation The concentration of CoPP used for grid preparation (1 mM) is high and raises concerns about potential artifacts. Such conditions may promote non-physiological intercalation or stacking of porphyrin molecules, as shown in Figure 4. The authors should comment explicitly on this limitation and discuss whether the observed stacking could reflect an artifact of sample preparation rather than a physiologically relevant transport state.
3. Mechanism of porphyrin/heme handling in vivo Freely diffusible CoPP (or heme) would be expected to equilibrate passively across membranes according to concentration gradients. Such a process would be inefficient, unregulated, and potentially hazardous, particularly because heme is a potent pro-oxidant capable of perturbing lipid organization and promoting lipid peroxidation. Because the study focuses solely on ABCB7 in isolation, whereas in vivo additional components such as FECH, ABCB10, and possibly other factors are present, the proposed model of lateral diffusion followed by capture by ABCB7 may be incomplete. A chaperone-mediated handoff mechanism seems more likely than bulk diffusion. In this context, ABCB7 could function as a sink or scavenger that prevents back-diffusion and enables vectorial transport. The authors may wish to discuss the possibility that heme transfer from mitochondria to the cytosol may be chaperoned in some of its steps.
4. Substrate specificity and structural evidence The authors show ATPase stimulation by hemin in Figure 1b. Do structural data exist for ABCB7 in complex with hemin, as opposed to CoPP? If not, this limitation should be acknowledged, particularly given the physiological relevance of heme relative to CoPP.
5. Relationship to ABCB10 When proposing that newly synthesized heme is handed off from FECH to ABCB7, the role of

ABCB10 should be discussed. Could the substrate-binding cavity of ABCB10 accommodate porphyrin analogs? Does ABCB10 contain a comparable arrangement of residues to those highlighted in Figures 2e-f for ABCB7? A brief comparative discussion would help place ABCB7 within the broader mitochondrial heme-handling network.
6. Overstatement of functional conclusions Lines 339–342 state that the findings of this study expand the role of ABCB7 to mediating heme export. However, the paper does not directly demonstrate heme transport. This statement should be toned down to reflect that the data support a potential or proposed role, rather than a demonstrated function.

Minor Comments
* Figure 1 legend: Although porphyrins are defined in detail in Fig. S2, they should also be briefly defined in the main figure legend for clarity.
* Figure 2d: Label residues G508 and S610 on the structure.
* Figure 5: ATP and Mg^{2+} are difficult to visualize and should be labeled more clearly.
* Figure 6: Residue E433 should be labeled more clearly.

Reviewer #2

(Remarks to the Author)
Human ABCB7 is a mitochondrial inner membrane transporter. Previously, it has been demonstrated that ABCB7 plays an essential role in cytosolic iron-sulfur cluster biogenesis and the regulation of intracellular iron homeostasis. In this manuscript, the authors present cryo-EM structures of human ABCB7 in combination with the binding of substrate (cobalt-protoporphyrin IX (CoPP)) and/or nucleotides, revealing the transition states from the apo-form inward-facing open conformation to the substrate-bound close conformation and the eventual modeled outward-facing conformation. The manuscript is well-written, and the detailed structural studies provided a molecular basis for how ABCB7 transports hemin across the mitochondrial inner membrane. Furthermore, the studies have identified three CoPP binding sites on the surface of ABCB7 within the bilayer membrane, which are presumably recruited for transportation via ABCB7.

Unlike ABCB6, ABCB7 is capable of binding two stacked CoPP molecules conjugated by two GSH. Within the binding cavity, the two CoPP-GSH have different interactions with ABCB7. Any cooperative nature to the binding of CoPP in ABCB7? What could be the physiological significance of two CoPP-GSH molecules in the binding cavity of ABCB7?

The authors proposed that CoPPs bound on the surface of ABCB7 within the bilayer membrane (Figure 4) will be laterally recruited to the binding cavity of ABCB7, but without further deliberation. For example, can GSH promote the transfer of these CoPP to the binding cavity in ABCB7? Under oxidative stress conditions, GSH is severely limited. Does this suggest that ABCB7 may not properly transport hemin under oxidative stress conditions due to the deficiency of GSH?

Reviewer #3

(Remarks to the Author)
In this paper, the authors have solved the structures of ABCB7 using cryo-EM, and they conclude that ABCB7 is a heme transporter, based on finding that one conformation of ABCB7 dimers accommodates two cobalt protoporphyrin IX molecules in a pocket formed by the dimeric transmembrane transporter. The paper is based on structure and is not supported by biochemical assays, and it therefore does not realize its full transformational potential. Also, the model they offer includes positing that heme is soluble in membranes, when this is an unlikely feature in the transition to heme exporter status, whereas it would make more sense for the transporter to draw from matrix non-membrane bound precursors, perhaps through mechanisms that ligate precursors at various steps and conformations. Their own experimental support for this possibility is not strong, and references from the literature, which has failed to resolve mysteries about ABCB7 for years, does not strengthen, as reported conclusions in the literature with respect to heme intermediates are often affected by older discredited models.

There are several aspects of writing, presentation and hypothesis generation that could be clarified and improved, which would allow the paper to constitute an important breakthrough in the analysis of ABCB7.

Points to consider:
1. In the abstract, they cite that ABCB7 is essential for cytosolic Fe-S cluster biogenesis, but the papers upon which this is based mostly discuss the yeast homologue, and this point, although often cited, has not been shown in other systems, although ABCB7 dysfunction clearly leads to iron overload in the mitochondrial matrix.
2. There is no strong support for their assertion that lipophilic porphyrins partition into the membrane and are funneled laterally into the translocation pathway in their model.
3. It would be preferable to leave the option that transient transport molecule interactions in the matrix side of ABCB7 transporters precede translocation into the central cavity.

Version 1:

Reviewer comments:

Reviewer #1

(Remarks to the Author)
The authors have addressed all my concerns. I'm satisfied with the revised version of the manuscript. I recommend careful re-reading to correct some typos as for instance at lines 275-277 ("in" should probably be deleted), and lines 390-391 (either "may" or "is" should be deleted).

Reviewer #2

(Remarks to the Author)
The authors fully addressed my concerns.

Reviewer #3

(Remarks to the Author)
The authors have addressed reviewer questions reasonably. The revised version is acceptable for publication.

Open Access This Peer Review File is licensed under a Creative Commons Attribution 4.0 International License, which permits use, sharing, adaptation, distribution and reproduction in any medium or format, as long as you give appropriate credit to the original author(s) and the source, provide a link to the Creative Commons license, and indicate if changes were made.
In cases where reviewers are anonymous, credit should be given to 'Anonymous Referee' and the source.
The images or other third party material in this Peer Review File are included in the article's Creative Commons license, unless indicated otherwise in a credit line to the material. If material is not included in the article's Creative Commons license and your intended use is not permitted by statutory regulation or exceeds the permitted use, you will need to obtain permission directly from the copyright holder.
To view a copy of this license, visit https://creativecommons.org/licenses/by/4.0/

Reviewers' comments:

Reviewer #1 (Remarks to the Author):

The authors show that the ATPase activity of ABCB7 is stimulated by Fe- and Co-protoporphyrin IX (PPIX) in the presence of GSH, leading to the proposal that ABCB7 functions as a metalloporphyrin exporter. Using cryo-EM, the authors determined structures of human ABCB7 in multiple conformational states at 2.3 Å resolution. These structures reveal a putative substrate-binding cavity accommodating two stacked CoPP molecules bridged by the thiol groups of two GSH molecules. A conserved phenylalanine residue is proposed to function as a molecular gate for substrate release. The authors further propose that stacked CoPP molecules (up to four) can partition into the lipid bilayer, transfer laterally into the ABCB7 translocation pathway, and be exported.

The study also offers insights into the pathogenic mechanism of the E433K XLSA/A variant, proposing that substitution of a negatively charged Glu with a positively charged Lys causes steric and/or electrostatic interference with substrate binding or release, given the polar nature of porphyrin substrates.

Overall, the paper is of interest and worth sharing with the scientific community. However, several important issues should be addressed to strengthen the conclusions.

Major Comments

1. Physiological relevance of substrate concentrations: Based on its similarity to ABCB6 and reported interaction with the heme biosynthetic enzyme FECH, the authors propose that ABCB7 functions as a porphyrin transporter. ABCB7 expressed in insect cells and reconstituted into nanodiscs exhibits a 1.5- and 3.7-fold stimulation of ATPase activity in the presence of hemin and CoPP, respectively, but only in the presence of GSH. A major concern is that the reported Km for CoPP (~16 microM) appears substantially higher than the expected concentration of heme or porphyrin intermediates in the mitochondrial matrix, which is generally thought to be in the low nM range. This discrepancy should at

least be discussed. One possibility is that ABCB7 accesses transiently elevated local substrate concentrations near the site of synthesis through direct or indirect interaction with FECH, but this remains speculative and should be clearly framed as such.

>> The K_m for CoPP reported in the original submission was obtained using a malachite green assay (Pi accumulation after 30 min). Such end-point measurements can overestimate apparent K_m because (i) the reaction may extend beyond the linear regime, (ii) ADP/Pi can accumulate and thus act as inhibitors of ATPase activity, and (iii) ATP can be substantially depleted during the reaction, all of which tend to collectively shift the dose–response curve to the right.

>> To minimize these artifacts, we attempted to re-measure ATPase activity using an NADH-coupled assay (initial-rate conditions) and refit the data. However, this approach was not feasible in the presence of porphyrins, as their strong absorption in the 320~450 nm range interfered with the 340 nm readout of the NADH assay *(Campanale et al. JBC, 2003, 278(30), 27354-61).*

>> We agree that the apparent K_m for CoPP measured *in vitro* exceeds the predicted low nanomolar concentrations of free heme reported *in vivo (Hanna et al. PNAS, 2016, 113(27), 7539-7544).* We therefore propose a plausible model in which, as the reviewer suggests, ABCB7 encounters transiently elevated local substrate concentrations through spatial coupling to ferrochelatase (FECH). In addition, ABCB7 has been reported to associate with a multimeric complex comprising FECH, mitoferrin-1 (MFRN1), ABCB10, and other partners *(Medlock et al. PLOS One, 2015, 10(8), e0135896).* Such assembly could promote substrate channeling, enabling efficient handoff of porphyrins with minimal bulk diffusion, thereby increasing local substrate availability. We have added these considerations on page 16 (lines 341-348).

2. High porphyrin concentrations used for cryo-EM grid preparation: The concentration of CoPP used for grid preparation (1 mM) is high and raises concerns about potential artifacts. Such conditions may promote non-physiological intercalation or stacking of porphyrin molecules, as shown in Figure 4. The authors should comment explicitly on this limitation and discuss whether the observed stacking could reflect an artifact of sample preparation rather than a physiologically relevant transport state.

>> We agree with the reviewer's concern. The reason that we used a high CoPP concentration was to maximize substrate occupancy within the ABCB7 cavity and obtain a well-defined

substrate density in cryo-EM reconstructions.

>> To directly assess whether the stacked density of CoPP in our structure reflects a concentration-dependent artifact, we prepared additional grids at 10-fold lower CoPP concentration (100 μM) and determined the substrate-bound structure at 3.1 Å resolution. Under these conditions, the stacking density of CoPP within the cavity was essentially unchanged, whereas the membrane-partitioned CoPP density was no longer detectable (Supplementary Figure 18). This suggests that the stacked porphyrin arrangement in the cavity is a robust structural feature of ABCB7 rather than an artifact of excess substrate. It further indicates that excess CoPP easily partitions into the lipid bilayer, where the hydrophobic environment stabilizes the porphyrin macrocycle and limits the aggregation or redox reactivity that is prone to occur in the case of free porphyrins in aqueous solution. We have added these points on page 17 (lines 364-369, 378-381).

3. Mechanism of porphyrin/heme handling in vivo: Freely diffusible CoPP (or heme) would be expected to equilibrate passively across membranes according to concentration gradients. Such a process would be inefficient, unregulated, and potentially hazardous, particularly because heme is a potent pro-oxidant capable of perturbing lipid organization and promoting lipid peroxidation. Because the study focuses solely on ABCB7 in isolation, whereas in vivo additional components such as FECH, ABCB10, and possibly other factors are present, the proposed model of lateral diffusion followed by capture by ABCB7 may be incomplete. A chaperone-mediated handoff mechanism seems more likely than bulk diffusion. In this context, ABCB7 could function as a sink or scavenger that prevents back-diffusion and enables vectorial transport. The authors may wish to discuss the possibility that heme transfer from mitochondria to the cytosol may be chaperoned in some of its steps.

>> We appreciate the reviewer's thoughtful insights and agree with the underlying concern. In response we now discuss the possibility that mitochondrial heme transfer is likely coordinated within multi-protein assemblies comprising heme biosynthetic enzymes, transport/accessory factors, and chaperone-like components *(Medlock et al. PLOS One, 2015, 10(8), e0135896)*. In this situation, ABCB7 may operate in close spatial coupling with FECH and other partners to facilitate substrate channeling during specific steps of heme biosynthesis. In the revised text,

we have removed the section proposing the lateral diffusion model and added these considerations to the Discussion (lines 404-415).

4. Substrate specificity and structural evidence: The authors show ATPase stimulation by hemin in Figure 1b. Do structural data exist for ABCB7 in complex with hemin, as opposed to CoPP? If not, this limitation should be acknowledged, particularly given the physiological relevance of heme relative to CoPP.

>> In addition to the CoPP:GSH dataset, we also collected cryo-EM data for ABCB7 in complex with hemin:GSH. Although the hemin:GSH-bound reconstruction reached 3.1 Å resolution, the hemin density appeared somewhat smeared, reflecting conformational heterogeneity and/or reduced occupancy. Nevertheless, the map supports a nearly identical binding mode: two hemin molecules occupy the same TM cavity in a stacked arrangement, and the associated GSH adopts a similar position, engaging the same set of key interacting residues. These data support a conserved binding mode for hemin and CoPP. We have addressed this point in the revised manuscript (lines 219-229, 382-391).

5. Relationship to ABCB10: When proposing that newly synthesized heme is handed off from FECH to ABCB7, the role of ABCB10 should be discussed. Could the substrate-binding cavity of ABCB10 accommodate porphyrin analogs? Does ABCB10 contain a comparable arrangement of residues to those highlighted in Figures 2e-f for ABCB7? A brief comparative discussion would help place ABCB7 within the broader mitochondrial heme-handling network.

>> Recent cryo-EM structures of human ABCB10 support its role as a biliverdin exporter and reveal a hydrophobic substrate-binding pocket with only limited positively charged patches (Cao et al., Nat. Commun. 2023, 14:2030). In addition, whereas ABCB7 uses GSH as a cofactor for substrate recognition, ABCB10 appears to engage cholesterol, further suggesting a substrate recognition mode distinct from that observed for ABCB7. Consistent with these structural features, sequence alignment indicates that the key residues forming the ABCB7-CoPP interaction network are not well conserved in ABCB10. Moreover, we confirmed that ABCB10 ATPase activity is not stimulated by hemin or CoPP under the conditions where ABCB7 is robustly activated (data not shown; manuscript in preparation). Collectively, the available

structural and biochemical evidence supports distinct substrate specificities for ABCB7 and ABCB10. We therefore discuss ABCB10 as a parallel component in mitochondrial heme metabolism, rather than as a transporter sharing its substrate-binding mechanism with ABCB7. Please see lines 392-403.

6. Overstatement of functional conclusions: Lines 339–342 state that the findings of this study expand the role of ABCB7 to mediating heme export. However, the paper does not directly demonstrate heme transport. This statement should be toned down to reflect that the data support a potential or proposed role, rather than a demonstrated function.

>> We agree with this comment and have revised the text.

- before

These findings expand the physiological significance of ABCB7 beyond its established role in maintaining mitochondrial iron and redox homeostasis to mediating the export of heme produced by ferrochelatase and linking that to the HO-1-dependent cytoprotective pathway.

- after

Expanding on the established functions of ABCB7 in maintaining mitochondrial iron and redox homeostasis, these results suggest novel roles for ABCB7 in heme export and the HO-1-dependent cytoprotective pathway.

Minor Comments

*** Figure 1 legend: Although porphyrins are defined in detail in Fig. S2, they should also be briefly defined in the main figure legend for clarity.**

>> We have revised the figure legend as suggested (lines 904-907).

*** Figure 2d: Label residues G508 and S610 on the structure.**

>> We have made the change as suggested.

*** Figure 5: ATP and Mg^{2+} are difficult to visualize and should be labeled more clearly.**

>> We have made the change.

*** Figure 6: Residue E433 should be labeled more clearly.**

>> We have made the change.

Reviewer #2 (Remarks to the Author):

Human ABCB7 is a mitochondrial inner membrane transporter. Previously, it has been demonstrated that ABCB7 plays an essential role in cytosolic iron-sulfur cluster biogenesis and the regulation of intracellular iron homeostasis. In this manuscript, the authors present cryo-EM structures of human ABCB7 in combination with the binding of substrate (cobalt-protoporphyrin IX (CoPP)) and/or nucleotides, revealing the transition states from the apo-form inward-facing open conformation to the substrate-bound close conformation and the eventual modeled outward-facing conformation. The manuscript is well-written, and the detailed structural studies provided a molecular basis for how ABCB7 transports hemin across the mitochondrial inner membrane. Furthermore, the studies have identified three CoPP binding sites on the surface of ABCB7 within the bilayer membrane, which are presumably recruited for transportation via ABCB7.

Unlike ABCB6, ABCB7 is capable of binding two stacked CoPP molecules conjugated by two GSH. Within the binding cavity, the two CoPP-GSH have different interactions with ABCB7. Any cooperative nature to the binding of CoPP in ABCB7? What could be the physiological significance of two CoPP-GSH molecules in the binding cavity of ABCB7?

>> Although ABCB7 accommodates two CoPP molecules simultaneously within the cavity, our data do not provide evidence for cooperative binding between them. Specifically, CoPP-

dependent ATPase stimulation follows a noncooperative model under the conditions tested (Hill slope ~1, Fig. 1c), and we do not observe hallmarks of cooperativity such as sigmoidal behavior.

>> Regarding the physiological relevance of the two CoPP:GSH binding sites, we propose that the unusually large cavity of ABCB7 may permit export of two cargo molecules per transport cycle, thereby increasing transport throughput when FECH-driven heme production is elevated. We have added this point in the revised manuscript (lines 350-354).

The authors proposed that CoPPs bound on the surface of ABCB7 within the bilayer membrane (Figure 4) will be laterally recruited to the binding cavity of ABCB7, but without further deliberation. For example, can GSH promote the transfer of these CoPP to the binding cavity in ABCB7? Under oxidative stress conditions, GSH is severely limited. Does this suggest that ABCB7 may not properly transport hemin under oxidative stress conditions due to the deficiency of GSH?

>> We thank the reviewer for raising these mechanistic questions. As shown in Fig. 4, free CoPP is highly hydrophobic and partitions strongly into the lipid bilayer. Consequently, the formation of a CoPP:GSH complex is expected to introduce hydrophilic functionalities to CoPP. This modification likely stabilizes CoPP once it enters the ABCB7 cavity, which is mainly lined with polar and charged residues. Thus, rather than being required for initial capture of substrate from the membrane, GSH may function as a cofactor that facilitates loading and stabilization of the substrate in the transporter cavity (lines 354-359).

>> Regarding oxidative stress, we agree that cellular GSH levels can be perturbed; however, mitochondrial GSH homeostasis is preferentially sustained through NADPH-dependent regeneration *(Hu et al., J Biol Chem 2008, 283(43), 29126–29134)* and the import pathway *(Liu et al., Science 2023, 382(6672), 820-828)*. Accordingly, we propose that, while ABCB7-mediated porphyrin transport may be attenuated under oxidative stress, it is not necessarily abolished. Please see lines 359-363.

Reviewer #3 (Remarks to the Author):

In this paper, the authors have solved the structures of ABCB7 using cryo-EM, and they conclude that ABCB7 is a heme transporter, based on finding that one conformation of ABCB7 dimers accommodates two cobalt protoporphyrin IX molecules in a pocket formed by the dimeric transmembrane transporter. The paper is based on structure and is not supported by biochemical assays, and it therefore does not realize its full transformational potential. Also, the model they offer includes positing that heme is soluble in membranes, when this is an unlikely feature in the transition to heme exporter status, whereas it would make more sense for the transporter to draw from matrix non-membrane bound precursors, perhaps through mechanisms that ligate precursors at various steps and conformations. Their own experimental support for this possibility is not strong, and references from the literature, which has failed to resolve mysteries about ABCB7 for years, does not strengthen, as reported conclusions in the literature with respect to heme intermediates are often affected by older discredited models.

There are several aspects of writing, presentation and hypothesis generation that could be clarified and improved, which would allow the paper to constitute an important breakthrough in the analysis of ABCB7.

Points to consider:

1. In the abstract, they cite that ABCB7 is essential for cytosolic Fe-S cluster biogenesis, but the papers upon which this is based mostly discuss the yeast homologue, and this point, although often cited, has not been shown in other systems, although ABCB7 dysfunction clearly leads to iron overload in the mitochondrial matrix.

>> The reviewer notes that the role of mammalian ABCB7 in cytosolic Fe–S cluster biogenesis has not been demonstrated as consistently as for Atm1. Nevertheless, Pondarré et al. have shown in a mouse model that loss of Abcb7 in the liver impairs cytosolic Fe–S cluster assembly *(Human Molecular Genetics, 15(6), 2006, 953–964).* Therefore, we would prefer to retain this point in the abstract.

2. There is no strong support for their assertion that lipophilic porphyrins partition into

the membrane and are funneled laterally into the translocation pathway in their model.

>> We have addressed this point in our response to Reviewer 1, comment #3.

3. It would be preferable to leave the option that transient transport molecule interactions in the matrix side of ABCB7 transporters precede translocation into the central cavity.

>>Please see the revised text (lines 413-415).
